# Machine Learning for Structural Health Monitoring of Aerospace Structures: A Review

**DOI:** 10.3390/s25196136

**Published:** 2025-10-04

**Authors:** Gennaro Scarselli, Francesco Nicassio

**Affiliations:** 1 Department of Aeronautics and Astronautics, University of Southampton, Building 176, Boldrewood Innovation Campus, Burgess Road, Southampton SO16 7QF, UK; 2 Department of Engineering for Innovation, University of Salento, Via per Monteroni, 73100 Lecce, Italy; francesco.nicassio@unisalento.it

**Keywords:** machine learning, SHM, aerospace structures, damage detection

## Abstract

Structural health monitoring (SHM) plays a critical role in ensuring the safety and performance of aerospace structures throughout their lifecycle. As aircraft and spacecraft systems grow in complexity, the integration of machine learning (ML) into SHM frameworks is revolutionizing how damage is detected, localized, and predicted. This review presents a comprehensive examination of recent advances in ML-based SHM methods tailored to aerospace applications. It covers supervised, unsupervised, deep, and hybrid learning techniques, highlighting their capabilities in processing high-dimensional sensor data, managing uncertainty, and enabling real-time diagnostics. Particular focus is given to the challenges of data scarcity, operational variability, and interpretability in safety-critical environments. The review also explores emerging directions such as digital twins, transfer learning, and federated learning. By mapping current strengths and limitations, this paper provides a roadmap for future research and outlines the key enablers needed to bring ML-based SHM from laboratory development to widespread aerospace deployment.

## 1. Introduction

The aerospace industry is built upon an uncompromising commitment to safety, performance, and reliability. Aircraft and spacecraft operate under harsh and variable conditions, including fluctuating pressures, extreme temperatures, mechanical vibrations, and aerodynamic loads. These stresses can lead to progressive damage such as fatigue cracks, delamination, corrosion, and other failure modes that, if left undetected, may compromise structural integrity. The significance of SHM has become increasingly apparent in recent decades. SHM encompasses techniques and systems for the real-time assessment of structural conditions through embedded or surface-mounted sensors, data acquisition units, and analytical methods. Its goals are to detect damage at early stages, inform maintenance decisions, and ultimately extend the service life of aerospace assets [1]. Several high-profile structural failures in civil aviation have highlighted the necessity of robust SHM. Notable examples include the 1988 Aloha Airlines Flight 243 incident, where undetected fatigue cracking led to explosive decompression mid-flight [2], and the 2002 China Airlines Flight 611, which disintegrated due to undiagnosed damage stemming from a prior tail strike [3]. More recently, the 2018 Lion Air Flight 610 and 2019 Ethiopian Airlines Flight 302 crashes, although primarily linked to software and sensor faults, have underscored the importance of integrated system health awareness, including structural aspects. These tragedies emphasize the critical need for advanced monitoring systems capable of capturing and interpreting complex structural behaviors in real time.

Traditional SHM methods, such as manual inspections, non-destructive testing (NDT), and model-based techniques, are often labor-intensive, time-consuming, and sometimes insufficient for capturing hidden or evolving damage. The growing complexity of aerospace structures, particularly with the use of composite materials and additive manufacturing, further challenges the limits of conventional approaches.

This review has two complementary goals: (i) to provide aerospace engineers with a concise introduction to the main ML methods, highlighting their principles, strengths, and limitations; and (ii) to critically assess the application of these ML techniques to SHM in aerospace structures. In this way, the paper aims to bridge the gap between the ML community and aerospace practitioners by offering a dual roadmap for methodological development and practical deployment.

## 2. Overview of Machine Learning

### 2.1. Terminology and Definitions

Machine learning (ML), a subfield of artificial intelligence (AI), enables algorithms to learn patterns directly from data rather than relying on predefined models [4]. In aerospace, the growth of sensor-rich structures has made manual analysis impractical, and ML now underpins applications ranging from anomaly detection to fatigue prognosis [5,6].

### 2.2. Models and Algorithms

ML approaches are commonly divided into supervised, unsupervised, deep learning, and hybrid learning categories.

**Figure 1:**
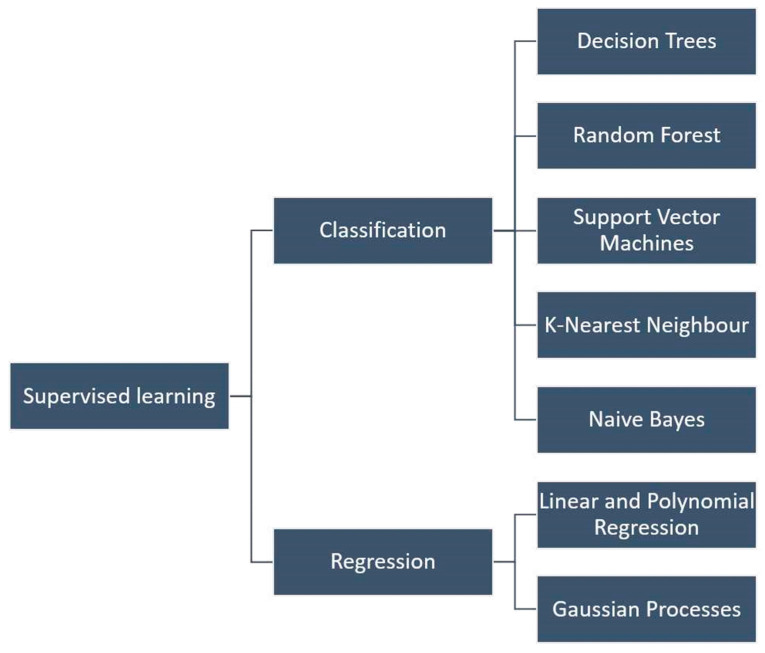
Supervised learning algorithms.

**Figure 2:**
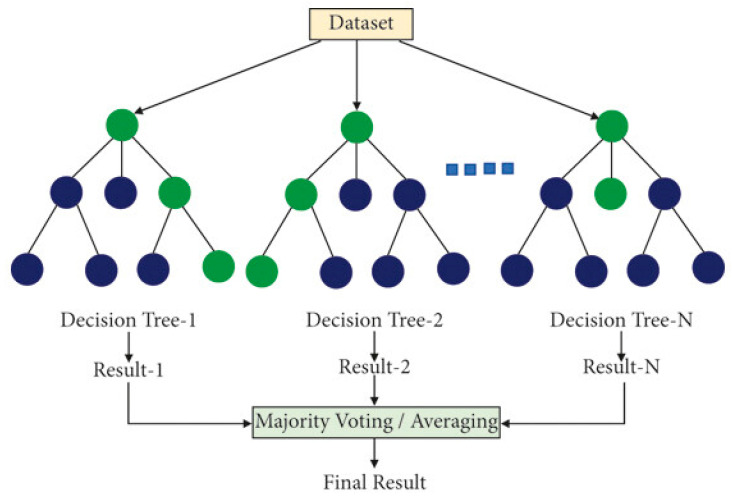
Random Forest architecture [9].

Supervised learning (see Figure 1) uses labeled data for classification or regression tasks [7,8,9,10,11,12,13]. Classical models include decision trees and Random Forests (Figure 2) [14,15], linear and polynomial regression [8,16,17], support vector machines (SVMs) [8,14,18], and K-nearest neighbors (KNNs) [8,14,19,20]. Bayesian methods and Gaussian processes extend supervised learning by quantifying uncertainty [14,21,22,23,24,25]. Their application to SHM is well established in strain-based detection and guided-wave monitoring [26,27,28,29,30]. A schematic of supervised learning principles is shown in Figure 1.

**Figure 3:**
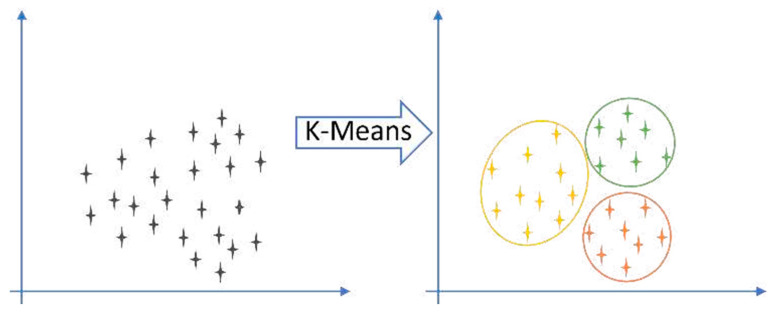
K-means architecture [7].

**Figure 4:**
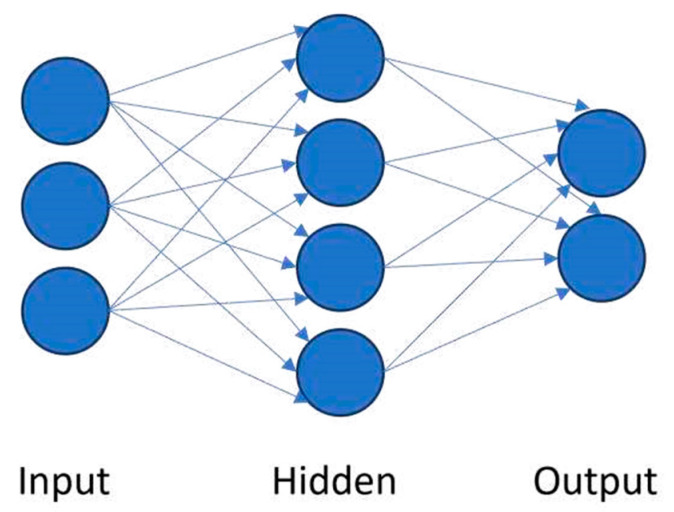
Artificial neural network layers [50].

**Figure 5:**
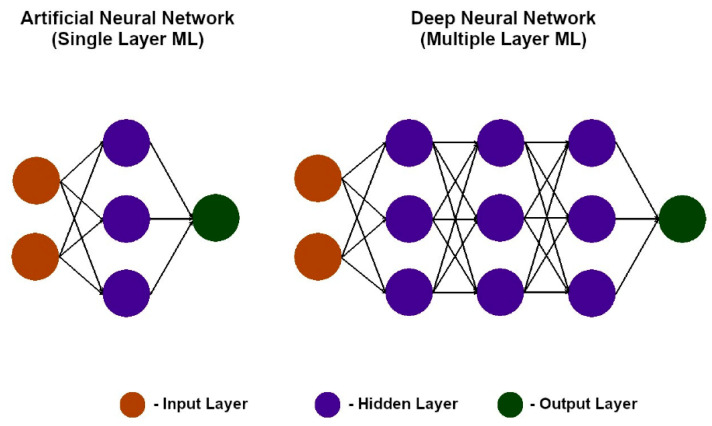
ANN vs. DNN architecture [62].

Unsupervised learning uncovers hidden structures in unlabeled data [8,14]. Clustering algorithms such as hierarchical clustering [31,32,33,34,35,36] and K-means (Figure 3) [34,37,38,39,40], together with dimensionality reduction methods such as principal component analysis (PCA) [41,42,43,44], have been widely applied to acoustic emission and guided-wave data to identify novel damage states [45,46,47,48]. Artificial neural networks (ANNs) provide flexible nonlinear modeling for regression, classification, and pattern recognition tasks (Figure 4) [49,50,51,52,53,54]. They perform well with large, complex datasets but are computationally demanding and less interpretable. Their advantages and limitations are summarized in Table 1, and Figure 5 illustrates the transition from shallow ANNs to deep neural networks (DNNs). While unsupervised learning is well suited for novelty detection in rare damage scenarios, the inherent class imbalance between abundant healthy data and scarce damage data remains a critical challenge in SHM. Semi-supervised approaches address this issue by exploiting large volumes of unlabeled or healthy data together with limited labeled damage samples, thereby improving sensitivity to rare events. Recent studies have applied semi-supervised variants of autoencoders and graph-based methods to detect delamination in composite laminates [55], to localize barely visible impact damage in CFRP structures [56], and to enhance guided-wave damage detection under imbalanced conditions [57]. Generative semi-supervised strategies, such as variational autoencoders and GANs, have also been employed to synthesize minority damage cases and rebalance training sets [58,59]. These approaches provide a practical compromise between the availability of operational flight data and the scarcity of representative damage examples, making them a promising complement to purely unsupervised novelty detection methods in aerospace SHM.

**Figure 6:**
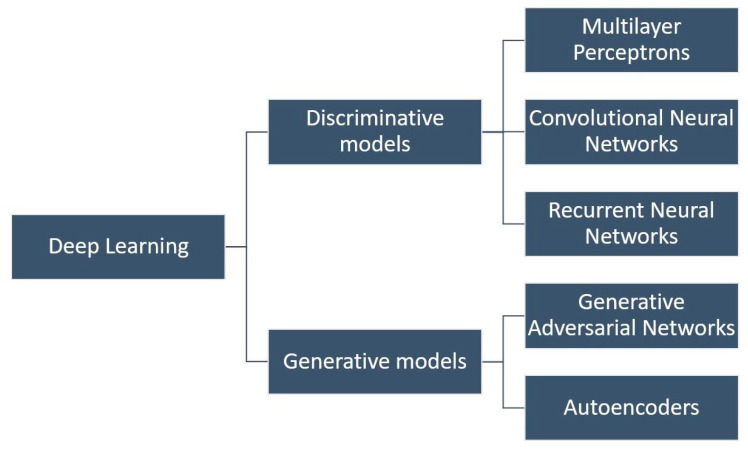
Deep learning algorithms.

**Figure 7:**
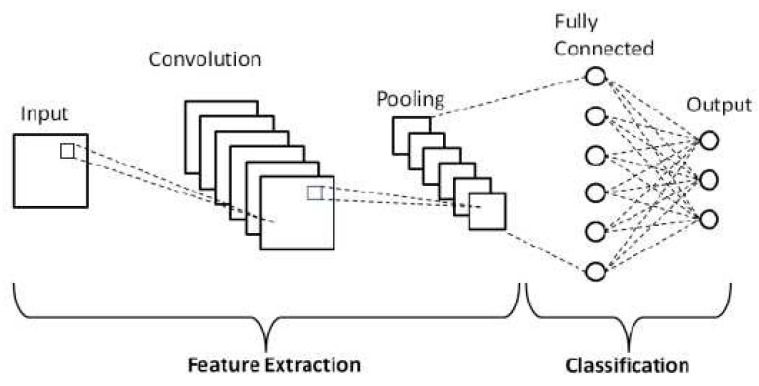
Convolutional neural network architecture [63].

**Figure 8:**
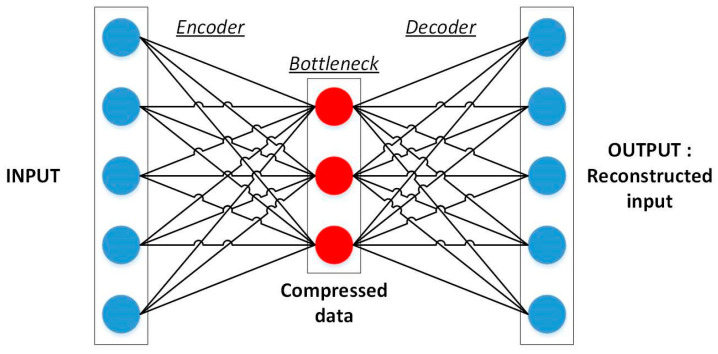
Autoencoder architecture [64].

Deep learning (DL) extends ANNs with multiple hidden layers, enabling automatic feature extraction from high-dimensional inputs such as images, vibration signals, or guided-wave spectrograms [60,61]. Major architectures include multilayer perceptrons, convolutional neural networks (CNNs, Figure 6 and Figure 7), recurrent neural networks (RNNs/LSTMs), generative adversarial networks, and autoencoders (Figure 8) [52,61,62,63,64]. DL models have shown promise in aerospace SHM, particularly for damage localization and fatigue prognosis [65,66,67,68,69,70]. Their advantages, limitations, and costs are summarized in Table 2.

Hybrid learning, often referred to as gray-box or physics-informed machine learning (PIML), combines physics-based models with data-driven approaches to leverage the strengths of both. This paradigm is particularly relevant to the SHM of aerospace structures, where experimental damage data are scarce and purely physics-based models may not capture complex operational variability [71,72]. The hybrid approach also introduced evolving physics-based material models enhanced by machine learning for multiscale composites by using physical sensing and digital twins [73,74]. Broader reviews have highlighted the importance of hybrid and physics-informed frameworks for aerospace applications, particularly in terms of reliability, certification, and uncertainty quantification [75,76,77].

### 2.3. Matching Aerospace Structural Problems with Machine Learning Algorithms: Principles and Trade-Offs

In the context of SHM, selecting a suitable machine learning algorithm is fundamentally driven by the type of structural problem, rather than the algorithm’s complexity alone. The characteristics of the data, such as the availability of labels, temporal consistency, and data dimensionality, also influence the algorithmic choice. Below, we describe typical problem types encountered in SHM and the rationale behind matching them to specific classes of machine learning approaches.

For **damage classification problems**, where different damage types are known and well-labeled, supervised learning algorithms (such as support vector machines or Random Forests) are generally appropriate. When damage patterns exhibit non-linear relationships or when the feature space is high-dimensional, artificial neural networks (ANNs) provide an effective modeling tool.

In **early-stage damage detection** or when damage scenarios are unknown or rare, unsupervised learning is a natural choice. These methods, including clustering and dimensionality reduction techniques, are designed to uncover deviations from a learned baseline of healthy behavior. Autoencoders, a bridge between unsupervised and deep learning, have gained traction for anomaly detection by compressing and reconstructing input patterns.

For **localization or severity quantification**, where the goal is to relate sensor data to physical variables like position or crack length, regression-based supervised models or deep learning architectures are appropriate. Convolutional neural networks (CNNs) and recurrent neural networks (RNNs) are particularly useful when dealing with spatial and temporal data, respectively.

**When raw, high-dimensional data, such as acoustic emission signals, vibration waveforms, or thermographic images, are involved**, feature engineering can be complex or impractical. In these cases, deep learning models are highly beneficial due to their ability to autonomously extract multiscale features from raw input.

Lastly, **long-term condition monitoring**, where the objective is to track progressive damage or fatigue over time, benefits from time-series models like RNNs or LSTMs, or unsupervised trend analysis approaches. These models capture temporal dependencies and can reveal subtle, gradual degradation that would be missed in static models.

Each class of algorithm brings its own strengths and limitations depending on the application context.

## 3. Overview of Structural Health Monitoring (SHM) for Aerospace Structures

According to Cusati et al., “Structural health monitoring represents an interesting enabling technology towards increasing aviation safety and reducing operating costs by unlocking novel maintenance approaches and procedures” [78].

In the 1980s, researchers started to study how SHM could be applied to aerospace structures and offshore platforms. SHM became established with the introduction of composite structures in the aerospace industry, which need to be continuously monitored and analyzed to predict and avoid any possible failure [1,4]. SHM makes it possible to meet this requirement, using sensor networks and machine learning algorithms. The main advantage of an SHM system is the possibility of performing online monitoring of the structure, in contrast to non-destructive testing (NDT), which requires an intervention plan to conduct the tests. Moreover, because of conventional NDT technology with inspections by specialized staff, the aircraft must be at rest, increasing operational costs. For these reasons, SHM is constantly developing and is therefore a matter of study in several fields of industry [79,80,81,82,83,84].

**Figure 9:**
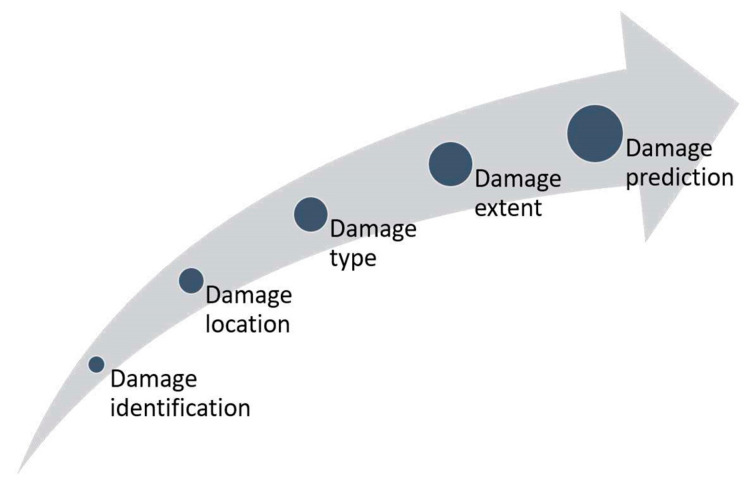
SHM levels.

An SHM system aims to identify structural damage to draw up a possible intervention plan. The process of damage identification can be summarized into three areas, namely detection, diagnosis, and prognosis, with a total of four levels, as established by Rytter [14]. As soon as ML has become more prevalent, a new level has been added for the detection of the damage type (level 3 in Figure 9) [84]. After detecting and locating damage, it is possible to classify and quantify it, to subsequently derive the residual useful life of the monitored structure.

SHM involves measuring several structural parameters to continuously analyze their variations over time. Therefore, it is necessary to acquire data, either from a numerical model or from experimental tests. There are three monitoring methods that can be employed:

- model-based;
- data-driven;
- hybrid.

The model-based approach involves the use of analytical and numerical models of the structure. The finite element method (FEM) is the most used numerical model, according to the literature [83,84].

This approach analyses the dynamic behavior of the examined structure and updates the model based on every variation of the evaluated parameters, which may be an index of damage presence [85]. For example, natural frequencies and mode shapes vary in the presence of damage; analyzing these kinds of parameters could lead to a robust damage identification model. This approach requires in-depth knowledge of the structure to be modeled, and it is complex to implement. Model-based approaches are further divided into two types of problems, inverse and direct. The former allows evaluation of the variation of the system parameters, starting from the measured data, while the latter allows evaluation of the perturbated parameters, starting from the damage characteristics.

As ML was introduced into the industry, SHM shifted from the model-based approach to the data-driven approach, which exclusively relies on the data collected by sensors, without the need for a structural numerical model. A statistical model implemented via algorithms analyses the data for damage prediction. Since this approach relies on statistical models, knowledge of the physical properties of the structure is not required: this approach can be suitable for complex structures [85]. For example, to perform a flight test, data is obtained from more than 200,000 sensors; more advanced ML techniques are being examined currently because big data has now become a reality in the aerospace industry [13].

In some applications, it is preferred to use a hybrid approach to support analysis through ML. Hybrid models consist of a combination of model-based and data-driven models to further improve the health monitoring process, as in [86].

### 3.1. Sensors

The core of a state-of-the-art SHM system is the sensor network, which enables the evaluation of defects, both during manufacturing and operational phases. The sensor network can be distributed over the structure to assess its global or local behavior. In a local SHM system, sensors are concentrated on the most critical points of the structure, such as the joints. In recent years, global–local approaches for localizing and quantifying damage have been developed by using, among others, ultrasonic guide waves and imaging algorithms, as studied by Balasubramaniam et al. [87].

**Figure 10:**
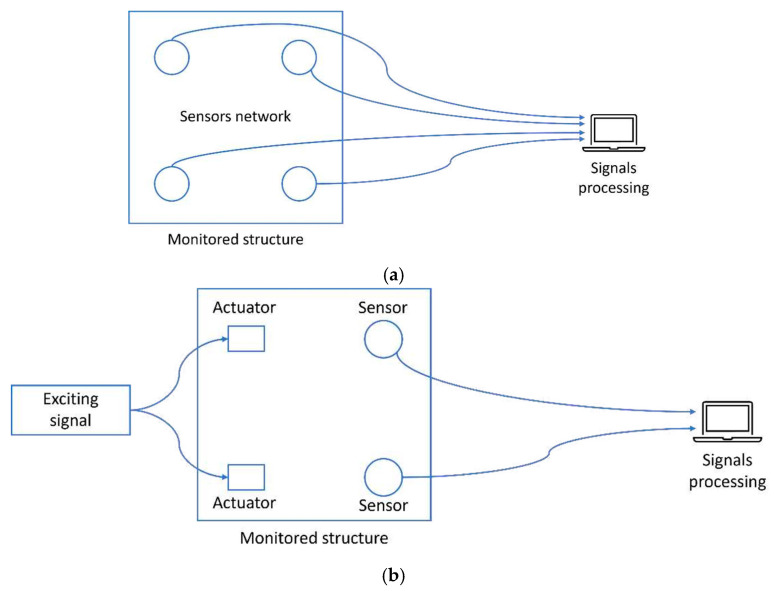
(**a**) Passive SHM vs. (**b**) active SHM [88].

Depending on sensor applications, an SHM system can be either passive or active [88,89]:

- In passive SHM, sensors are used to detect any change in the physical properties of the structure under examination, “listening” to the structural dynamical behavior (Figure 10a);
- In active SHM, the structure is triggered via actuator sensors, and its response is detected via receiving ones (Figure 10b).

In recent years, a study by Saeedifar et al. [90] analyzed active and passive SHM methods for impact damage assessment on a carbon fiber-reinforced polymer (CFRP) composite plate. Dipietrangelo et al. [91,92] presented ML algorithms to detect impacts on and damage to aeronautical aluminum plates via a passive approach.

Depending on the specific application, various types of sensors can be used, including piezoelectric sensors, fiber optic sensors (FOSs), eddy current sensors, resistance strain gauges, air/vacuum galleries, and nanomaterials [93]. The most common sensors in the aerospace field are

- fiber optic sensors, used to monitor the health of structures, as they can detect changes in strain (e.g., fiber Bragg gratings (FBGs));
- piezoelectric sensors, used to detect damage, both globally and locally (e.g., piezoelectric lead zirconated titanate (Pb[Zr_x_Ti_1−x_]O_3_ (0 ≤ x ≤ 1), PZT)).

#### 3.1.1. Fiber Optic Sensors

**Figure 11:**
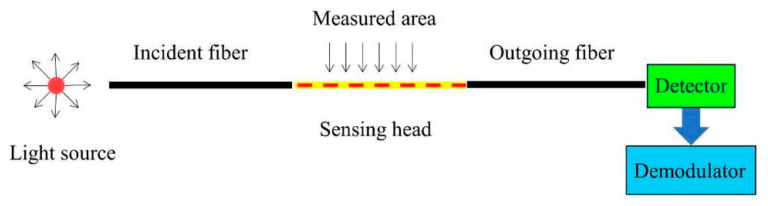
Fiber optic working principle [94].

Fiber optic sensors can be identified into three main classes: interferometric, grating-based, and distributed. The operating principle of an optical fiber (Figure 11) is based on the interaction between light and fibers: a light source is fed into the fiber and transmitted to the sensing head, where light modifies the optical properties (such as intensity, phase, wavelength…). The signal is then received by the detector and demodulator to obtain the measured parameters [94].

**Figure 12:**
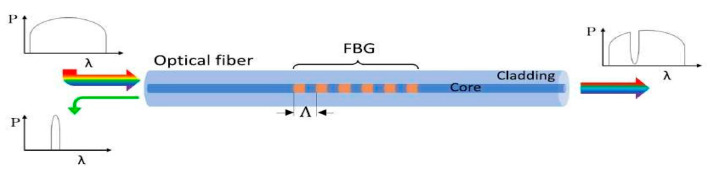
Operating principle of an FBG [97].

According to the technical literature for the aerospace sector, the most used fiber optic sensors are fiber Bragg grating (FBG) sensors because of their multiplexing capability: several Bragg gratings, which are independent of each other, can be installed on a single optical fiber [95], with the advantage of being immune to electromagnetic interference. As fiber optic sensors are employed to monitor the state of structures’ health, FBGs are utilized to measure the strain state of the structure to which they are attached [96]. A schematic representation of an FBG is shown in Figure 12.

**Figure 13:**
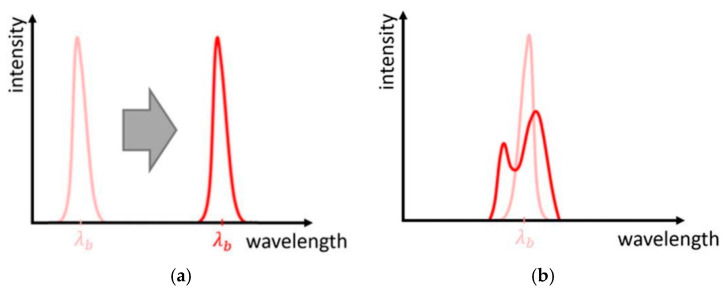
(**a**) Bragg wavelength shift; (**b**) Bragg peak distortion [96].

When light (i.e., UV or laser) penetrates an optical fiber and encounters the Bragg grating, the portion of light traveling at the Bragg wavelength *λ_B_* is reflected by the grating (left portion of Figure 12). The remaining portion of light continues its path (right portion of Figure 12). The output is then a spectrum in which the reflected light is missing. The phenomenon is governed by the Bragg equation:

(1) $$\lambda_{B}\;=\;2n_{eff}\Lambda$$

where *n_eff_* represents the effective fiber refractive index and Λ represents the perturbation period. The Bragg wavelength depends on strain and temperature. Therefore, in the presence of mechanical or thermal loads, grating deformations occur, perturbation period varies, and Bragg wavelength shifts (Figure 13a). In addition, Bragg peak distortion might also occur due to non-uniform loads applied to the grating (Figure 13b) [96].

A limitation of FGB sensors is that any change in temperature makes it difficult to detect a correct change in strain [98]. This issue is emphasized in aircraft structures, as the operating temperature range varies between −55 °C and 40 °C: consequently, it is necessary to perform temperature compensation [99,100,101,102,103].

#### 3.1.2. Piezoelectric Sensors

Piezoelectric sensors are widely used for SHM applications in aerospace due to their small size and weight, low cost, availability in various formats, and high sensitivity.

The piezoelectric sensors operating principle is based on the “piezoelectric effect”, discovered by the Curie brothers in 1880: when an external force is applied to certain dielectric crystals in a specific direction, the crystal tips create the same quantities of positive and negative charges. The density of these charges is proportional to the applied stress, and this is called the piezoelectric effect. It was later confirmed that an inverse piezoelectric effect exists, whereby the piezoelectric material, under a given electric field, produces a deformation of its crystal lattice (which is restored when the electric film is removed). Therefore, piezoelectric sensors can be used as both sensors and actuators [104].

Piezoelectric materials can be divided into three categories [104]:

1. Inorganic materials, which are subdivided into piezoelectric single crystals, piezoelectric ceramics, and piezoelectric films. Among these, PZT is the one with the best performance.
2. Organic materials, mainly piezoelectric polymer, which are employed in flexible sensors.
3. Composite materials, developed to improve the piezoelectric properties of organic materials by blending them with inorganic materials.

Piezoelectric sensors are mainly used for damage detection. Their use is common in the following technologies:

- Guided-wave and ultrasonic propagation (active);
- Electro mechanical impedance (EMI) (active);
- Acoustic emission (passive).

#### 3.1.3. Smart Structures

Sensors can be mounted onto the structure’s surface or inside the structures. Surface sensors (SSs) have several disadvantages [105]:

- Sphere of influence monitoring is primarily local;
- Signal interference with external factors leads to measurement uncertainty;
- Wiring is required, which can deteriorate, limiting large-scale use, causing electromagnetic interference and adding weight.

Wireless technology has developed over the years to overcome these issues.

Embedded sensors (ESs) address the drawbacks of the previous ones, with the so-called “smart structures”, where sensors are placed inside the structure to [105] (i) protect them from environmental factors, (ii) improve aerodynamic performances, and (iii) enhance the coupling sensors’ structure. These sensors must possess the following characteristics [106]:

- Low weight and compact dimensions;
- Continuous operational working;
- High sensitivity.

ESs (particularly FOSs) can also be used in the production phase of composite materials by inserting them within the matrix or between laminates to monitor resin flow, fiber alignment, laminate stacking sequence, and curing. Additionally, they monitor residual stresses and temperature both during production and, subsequently, during operation [89].

There are limitations/cons to the use of FOSs [107], such as

- Incorrect installation could lead to their own breakage;
- Composite fiber orientation influences the spectral FOS response;
- Optical fiber interleaving between composite fibers implies a defect that leads to the matrix breaking and structure delamination.

Meanwhile, PZT sensors are less suitable than FOSs for monitoring during the production of composite materials, since they are influenced by Curie temperature. These sensors also have a brittle nature (being primarily composed of ceramic materials) and less than ideal compatibility with flexible composite materials. Moreover, embedding PZT sensors leads to some limitations because of their volume, leading to delamination and a consequent strength reduction [107]. Additionally, they need to be shielded because they are sensitive to electromagnetic interference. Therefore, research has shifted towards other types of materials, such as “flexible materials”, which enhance the depolarization temperature [108], and composite piezoelectric sensors (PFCS), as studied by Konka et al. in [109].

To overcome all these limitations, nanotechnology and materials research is advancing. Regarding polymers, Tuloup et al. [110] analyzed the use of a new embedded sensor, namely polyvinylidene fluoride (PVDF), which is a flexible and extensible polymer. According to the authors, in some SHM applications, PVDF is preferred over traditional PZT sensors due to its lower fragility. Another material currently under research is MXene, a family of 2D nanomaterials with a layer of carbide or nitride (X layer) sandwiched between transition metal layers (M layer). Grabowski et al. [111] demonstrated that the response times are comparable to piezoelectric sensors, making it an excellent candidate for future multifunctional sensor developments. Zhang et al. [112] used a flexible MXene/CNT (carbon nanotube) film sensor, showing that it can be used throughout the composite lifecycle, starting from the production phase (e.g., monitoring of resin properties during the curing phase). Chen et al. [113] demonstrated that a mesh-structured piezoresistive sensor provides better compatibility with the host material, and mechanical properties are not affected, unlike what might occur with traditional sensors. The use of multiple sensor types should not be ruled out to combine the advantages of each. A hybrid sensor network of PZT/FBG was employed by Yu et al. [114] to monitor the curing cycle parameters of CFRP composites. The authors demonstrated that in addition to the production phase, the entire lifecycle of CFRPs can be effectively monitored using this kind of sensor network.

A major trend emerging in recent years is the integration of wireless ML-driven monitoring with advanced composites. Negoita et al. [115] presented a CFRP coupon study in which embedded carbon nanotube piezoresistive sensors transmitted strain and load data wirelessly to a DNN predictor. Their system achieved real-time prediction of mechanical properties (MAE = 0.14) with sub-second latency, demonstrating the feasibility of in-flight SHM linked to digital twins. This approach eliminates wiring complexity, reduces weight, and is directly relevant to composite airframes, where embedded sensing is a long-term goal.

### 3.2. Data Collection

The effectiveness of an SHM system relies not only on sensor type but also on how sensor data are collected, processed, and analyzed. A rigorous description of these steps is essential to understand the path from physical measurement to actionable structural information.

#### 3.2.1. Deployment

Sensor deployment in aerospace structures requires careful consideration of weight, aerodynamics, and accessibility. Surface-mounted sensors are easier to install and replace but add wiring complexity and are more exposed to environmental degradation. Embedded or in situ sensors, in contrast, provide better coupling with the host material and protect against environmental factors, but raise challenges in terms of manufacturability, structural integrity (risk of delamination), and certification. In practice, hybrid layouts are often adopted: sparse global arrays capture overall structural behavior, while dense local clusters are deployed near critical regions, such as joints, stiffeners, or cut-outs. Recent studies also highlight the role of wireless and self-powered sensors in reducing cabling and weight penalties, an important consideration for large composite airframes.

#### 3.2.2. Data Acquisition

Raw signals may take the form of strains, displacements, accelerations, or acoustic/vibration waveforms. High-fidelity data acquisition typically requires sampling frequencies from several kHz (strain, FBG) up to tens of kHz or higher (ultrasonic Lamb waves, acoustic emission). For instance, in aero-engine bearing monitoring, up to six sensors—two eddy-current probes for rotor displacement and four accelerometers on the casing—have been deployed, recording 15 s sequences at 25 kHz [116]. These details highlight the importance of sensor selection, number, orientation, and placement geometry in shaping the SHM dataset.

#### 3.2.3. Preprocessing and Signal Conditioning

Before analysis, signals are denoised and normalized to mitigate environmental and operational variability. Common preprocessing includes filtering, fast Fourier transform (FFT), wavelet decomposition, or empirical mode decomposition (EMD). EMD, for example, decomposes vibration signals into intrinsic mode functions, which can be selectively analyzed to highlight defect-related frequencies [116]. For guided-wave methods, baseline subtraction or temperature compensation is routinely applied to account for environmental changes.

#### 3.2.4. Feature Extraction and Representation

From pre-processed data, features are derived in the time domain (peak, RMS, kurtosis), frequency domain (spectral peaks, harmonics), or time–frequency domain (wavelet coefficients, spectrograms). More recent approaches adopt representation learning: converting time series into 2D images (e.g., Gramian angular fields) or constructing relational models such as spatial–temporal hypergraphs [116]. These frameworks capture both correlations across multiple sensors (spatial edges) and temporal dynamics (temporal edges), enabling models to exploit the structural interdependence of signals.

#### 3.2.5. Analysis and Model Training

Collected features feed into machine learning pipelines for damage detection, localization, or prognosis. Training involves dividing the dataset into training, validation, and test partitions; cross-validation or transfer learning may be employed to ensure generalization. Evaluation metrics go beyond accuracy, incorporating confusion matrices, precision, recall, F1-score, and robustness under varying operational conditions. Advanced studies also perform ablation analyses to quantify the contribution of each sensor type, feature, or network module to final performance [116].

#### 3.2.6. Challenges

While sophisticated sensor networks can generate large, high-dimensional datasets, aerospace constraints (weight, wiring, environmental robustness) limit the number of deployable sensors. Embedding wireless nanomaterial-based sensors or hybrid FOS–PZT arrays is one solution, but these approaches necessitate advanced data fusion and adaptive algorithms to handle heterogeneous data streams.

## 4. ML Applications for SHM on Aerospace Structures

### 4.1. SHM Methods

There are several methods that allow detection of any change in the structure response, with respect to its healthy state (any variation of properties and/or behavior of a structure can be associated with the presence of damage). According to the literature, the methods most used in aerospace are analyzed in the following subsections.

#### 4.1.1. Guided Waves and Ultrasonic Propagation

This technology employs ultrasonic or guide waves, such as Lamb and Rayleigh waves, emitted by a transducer (or actuator) and collected by sensors. Lamb waves differ from Rayleigh waves based on the analyzed structure. The former are employed in thin structures, while the latter are used in thick structures. Since aerospace structures are plate-like, the most used waves are Lamb waves. Lamb waves are divided into two modes, antisymmetric A_n_ and symmetric S_n_, based on wave frequency. The typical frequency band for thin plates is in the order of hundreds of kHz; hence, only A_0_ and S_0_ modes arise [117,118].

**Figure 14:**
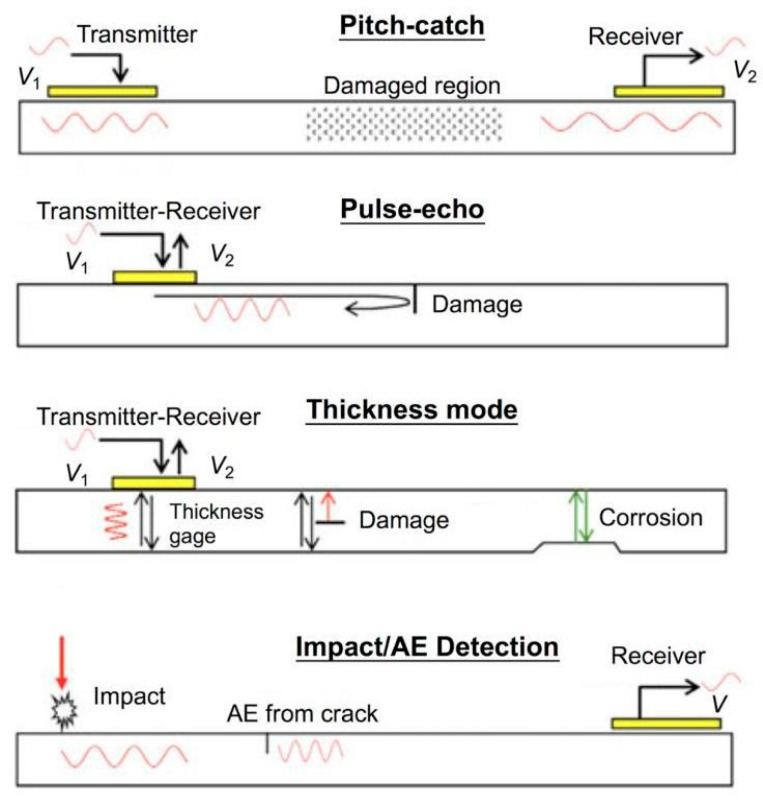
Guided-wave and ultrasonic propagation methods [104].

PZT sensors are suitable for this application since they can be used as both actuators and sensors. This method can work in different configurations (Figure 14, [104]), such as

- Pitch-catch, in which there are two sensors, one acting as an actuator to propagate the ultrasonic wave that passes through the structures and reaches the other sensors. The time-of-light (TOF) varies in the presence of a defect.
- Pulse-echo, in which there is only one sensor, which sends the guided wave and receives the reflected one in the presence of a defect.
- Thickness mode, in which the sensor excites the structure in the thickness direction. Changes in thickness, due to corrosion and/or damage, are detected.
- Impact/AE detection, in which the sensor receives a guide acoustic wave during an impact.

#### 4.1.2. EMI Acoustic Emissions

This method is based on the mechanical properties of piezoelectric sensors and sensor–structure coupling. It employs an actuator, which converts the electrical signal into mechanical stress, and a sensor, which converts the structural response into an electrical signal [104]. Any damage results in a change in structural stiffness and, consequently, in relative resonance properties. Due to electromechanical coupling, there is a variation in sensor impedance [93]. The real-time measured values are then compared to those recorded during the lifetime of the structure, to detect any variations that indicate the damage presence.

#### 4.1.3. Vibration-Based

This method allows for the detection of the variation in the structural vibrational response, mainly focusing on natural frequencies and mode shapes. This technique can be implemented via piezoelectric sensors or accelerometers. The advantages include the fact that few sensors are needed, and it is not necessary to know the damage location beforehand. However, modeling (mainly through FE models) of the structure under examination is necessary to conduct modal analysis [119,120].

#### 4.1.4. Strain-Based

Strain gauges have been overcome by fiber optic sensors, especially FBGs, in the measurement of strain in SHM applications. The authors of [121] developed a method to optimize FOS placement to detect strain variation. The strain measurements along the zero-strain trajectory (ZST) are considered a damage index. If the strain state of the structure changes, the measured strain along the ZST goes from zero to a specific value [122]. Table 3 illustrates some research articles that studied the different monitoring methods to guarantee the structural reliability of composite materials.

Fan et al. [123] proposed manifold learning approaches (e.g., Diffusion Maps combined with autoencoders) to compress and denoise guided-wave data under varying operational states. Their method improved both anomaly detection and state estimation across temperature and loading changes. This directly addresses one of the most persistent challenges in aerospace SHM—the sensitivity of Lamb-wave methods to environmental and operational conditions (EOCs).

**Table 3:** Studies on monitoring methods employed for composite structures.

Monitoring Method	Sensors	Research Articles
Guided-wave/ultrasonic propagation	Piezoelectric	[124,125,126]
EMI	Piezoelectric	[127,128]
Acoustic emissions	Piezoelectric	[129,130,131]
Vibration-based	Piezoelectric/accelerometers	[132,133]
Strain-based	Fiber optic/strain gauges	[134,135,136]

The following sections outline the research studies that have been conducted in recent years to understand how to reduce the risk of damage from impact and fatigue, leading to composite delamination and therefore the probable failure of the structure. This analysis includes principal research studies that use machine learning algorithms to ensure that the examined structure remains in optimal condition. A search of research articles (excluding reviews, theses…) ranging from 2019 to 2025 was conducted to present a state of the art of machine learning algorithms applied to structural health monitoring for aerospace composite structures. All the collected articles were divided into four categories: (i) damage diagnosis (detection, localization, classification, and quantification), (ii) fatigue prognosis, (iii) impacts detection, and (iv) others.

### 4.2. Damage Diagnosis

#### 4.2.1. Detection

According to Rytter, the first level of structural health monitoring is damage detection (see Table 4).

Alvarez-Montoya et al. [26] utilized an FBG sensor network to collect strain data from 16 flight tests—6 conducted with a healthy structure and the remainder with artificially simulated damage—to detect damage on the wing’s front spar of a UAV made of a composite balsa core/CFRP skin sandwich. A wireless system allows the transmission of sensor data to the ground, where Self-Organizing Map (SOM) and PCA algorithms are employed to cluster the operating conditions and identify damage. The method is found to be robust toward different environments and operating conditions and achieves an accuracy of 98.1%, making it possible to be used for remote monitoring of flying structures, thus promising savings in time and maintenance costs.

Strain measurements are also used in [27] to detect damage. In particular, the authors employ an embedded FOS in a carbon/epoxy plate, subjected to different four-point bending tests, to create a strain-based database. An ANN is trained on the healthy structure data and predicts damage based on any correlation differences between the strains measured in a specific area of the plate. The method can be generalized to any structure (since it is independent of the material type) and to any load condition. The results show a high resolution in damage detection.

In [28], two features (namely ToF and signal attenuation) are extracted from ultrasonic wave signals emitted in a composite panel to generate a training database for a Bayesian optimization algorithm. The proposed approach is based on a novelty index of the measured data: inequalities between measured points to detect anomalies indicative of the presence of damage. The algorithm stops when it no longer receives data or detects damage, and it minimizes the number of observations required, speeding up the damage detection phase, and consequently reducing time and computation costs.

In addition to data-driven approaches, it is possible to use model-based approaches for damage detection. Bergmayr et al. [29] utilize an FEM model to simulate a Nomex/GFRP composite sandwich aeronautical spoiler. The FEM model allows for extracting strain data for the healthy structure, and then for the damaged one, by statistically modifying the previous values. A dataset is then created to train a multilayer perceptron (MLP) network, which can classify the structure’s state of health. The method is then validated through experimental tests by recreating the spoiler structure, drilling a hole to simulate damage, and subjecting it to different loads. The numerical and experimental results are satisfactory, demonstrating good efficiency in damage detection in composite sandwich structures.

Tran-Ngoc et al. [137] propose an efficient ANN to detect damage in composite laminates, modeled via the FEM model. Natural frequencies are fed as input into the network. A vectorization technique is employed to reduce the dimensionality of the data. A hybrid metaheuristic optimization algorithm enables optimization of the ANN guarantying the network to find the optimal solution. The proposed method can achieve high accuracy with respect to traditional ANNs.

These examples highlight how classical supervised learning methods (Section 2.2), such as SVMs and Random Forests, remain highly effective for known damage states, while unsupervised strategies (Section 2.2) enable novelty detection when labeled data are scarce. Yet, while high accuracies are consistently reported, differences in performance stem from fundamental methodological trade-offs. Supervised approaches deliver strong results for well-characterized datasets, but are limited to pre-defined damage types and often underperform in unlabeled or rare-damage scenarios. Unsupervised methods (e.g., PCA, SOM) excel at novelty detection without the need for labels, though their outputs are less interpretable and more prone to false alarms under environmental variability. Model-based FEM–ML hybrids bridge this gap by embedding physical knowledge and reducing data requirements, but their reliability depends heavily on model fidelity and assumptions. In aerospace contexts, where real damage labels are scarce and operating conditions vary significantly, these trade-offs are more decisive than raw accuracy percentages.

#### 4.2.2. Localization

The second Rytter level involves damage localization. Various approaches for achieving this purpose are outlined in the following research articles (see Table 5).

In [65], a CNN enables localization of damage in a CFRP composite plate using guided waves. The Piecewise Aggregate Approximation (PAA) algorithm processes the extracted signals, while the Gramian Angular Field (GAF) algorithm converts the signals into 2D images. After the training phase, the CNN model automatically extracts damage-related features and provides the coordinate vector of damage in the CFRP plate. The results show an error of 7.58% in damage localization, indicating that the method achieves good performance.

Song et al. [138] utilize the Global-Local Feature Cross-Fusion Network (GLFCFN) method to localize damage in a composite panel. This method employs four PZT sensors to emit ultrasonic waves throughout the structure and a DL approach to localize damage. The method starts with feature extraction from ultrasonic signals, based on 1D-CNN algorithms, for local damage information, and gated multilayer perceptron (gMLP) for global information. A method based on the “multi-head attention” mechanism allows the extracted features to be fused to analyze damage information, providing a better damage position prediction. The GLFCFN method is experimentally validated and proves to have better performance than other traditional methods.

In [139], the EMI method and a Modified Probabilistic Damage Imaging (MPDI) algorithm are employed on a Nomex/T300 CFRP composite sandwich to detect damage location. The damage is artificially simulated by inserting a small piece of wave-absorbing material near two sensors. Features are extracted from signals using the Direct-Coupled Mechanical Impedance (DCMI) methodology and root mean square deviation (RMSD). The MPDI algorithm allows detection and visualization of damage position, with high accuracy, as experimentally demonstrated.

Similarly, in [140], the authors utilize an imaging algorithm in the dictionary learning context to create a damage localization method for complex composite structures. A movable magnet is used to simulate damage on a carbon/epoxy plate, where an array of PZT sensors generates Lamb waves for the training phase. The developed imaging algorithm is based on sparse reconstruction (SR), and experimental results prove its ability to detect the damage position, with a small radial error. To assess the effectiveness of the algorithm in impact scenarios, an experimental test campaign is conducted on the same plate, and the proposed method demonstrates better performance than other learning methods despite some localization errors.

In the study [141], Lamb waves are emitted throughout a CFRP composite plate, where superficial damage is simulated by placing different diameter masses at various points. Subsequently, via a low velocity impact, interlaminar damage is simulated. After signal acquisition, the model utilizes a deep convolutional neural network (DCNN) to extract features, and a recurrent regression algorithm to localize damage. According to the results obtained, the method achieves high accuracy and resolution for damage localization, requiring only a few sensors. Moreover, the method can be extended to the multi-point cumulative damage localization of composite structures.

There are many studies that analyze both first and second levels. Sawant et al. [45] utilize ultrasonic waves for damage detection and localization on a CFRP composite panel. Unlike the previous study, the authors employ a convolutional autoencoder (CAE), which does not require signal pre-processing. Furthermore, the authors utilize the transfer learning (TL) technique to reduce the number of training parameters to up to 95%, without affecting the accuracy of the method. The proposed TL-CAE framework is then validated on the public Open Guided Waves (OGW) database. The results demonstrate that the method is scalable to any material, since pre-processing of signals and prior knowledge of the material are not required.

An identification method, based on both levels, is developed by Rautela et al. in [66], for a CFRP composite panel. Ultrasonic signals are pre-processed via experts’ knowledge, prior to the training phase of a CNN model. Two convolutional neural networks are employed, respectively, for damage detection and localization. In particular, the first network establishes the health state of the structure, namely healthy or damaged, whereas the second network activates damage localization if the output of the first network is the damaged condition. The first network reaches an accuracy of 99.1%, while it is 99.3% for the second network. An advantage of this study is the reduction in the number of sensors employed in the offline training phase, allowing a weight reduction of the monitored structure.

In the study [142], a probabilistic imaging algorithm is employed on different case studies for a CFRP specimen: (i) healthy specimen, (ii) specimen with different damages. Multipath Lamb waves are emitted by PZT sensors throughout the specimens, and the algorithm detects and localizes damage by evaluating the wave path deviation after the reflection, as demonstrated in case (ii), while the wave path remains the same in case (i). The advantage of using the multipath Lamb-wave technique is that it allows the number of transmitter–receiver couples to be reduced, unlike for single-path Lamb waves, since a larger area of the structure can be monitored with few sensors.

In contrast to previous studies, research in [30,143,144,145] use a model-based approach to detect and locate damage. In [30], the behavior of a Nomex/GFRP composite sandwich aeronautical spoiler is simulated by means of the FEM approach. Both the healthy and damaged structures are modeled, by considering a hole in the spoiler, to evaluate strains under different load conditions. Strain data derived from the healthy structure are used to train a Random Forest (RF) classifier. Healthy strain data are then statistically modified to simulate damaged data of the damaged structure. The RF classifier can achieve good performance when it is tested on numerical data, while performance is slightly worse when it is tested on experimental results.

A model-based approach is also used by the authors of [143] to detect and localize damage in CFRP pin-joined truss structures. An FEM model generates vibrational data for both healthy and damaged structures as input for a hierarchical CNN model. Damages are simulated considering compromised bolted connections. The numerically trained CNN is then validated on numerical data, demonstrating that the proposed algorithm can be generalized. The proposed method is then compared with a non-hierarchical classifier, which is not able to generalize experimental data.

The authors of [144] utilize a Spectral Finite Element Method (SFEM) model of a carbon fiber/epoxy laminate to generate two datasets, respectively, in time and time–frequency domains. Two deep neural networks, 1D-CNN and 2D-CNN, are trained on these datasets and are used to classify the health state of the structure, i.e., healthy or damaged. 2D-CNN trained on the time–frequency domain proves to be better than the others. A CNN and a Long Short-Term Memory (LSTM) recurrent neural network are employed to localize damage. These two algorithms demonstrate to achieve high performance.

Lin et al. developed, in [145], an FEM model of the Airbus A350 composite wing to numerically simulate its behavior under different operating flight conditions. Healthy and damaged numerical data are used to train a CNN model by simulating different damage sets. The classification of the health state of the structure (healthy or damaged) reaches an accuracy of 99% without noise and an accuracy of 97% with 2% of Gaussian noise. Regarding the localization task, to establish the performance of the model, tests are carried out for different threshold values (THR), which are strictly related to the error tolerance between the actual location of the damage and the predicted ones by the algorithm.

The reviewed studies (see Table 6) demonstrate the relevance of deep learning architectures (Section 2.2), particularly CNNs and RNNs, for extracting spatial and temporal features from guided-wave data, while hybrid FEM–ML approaches reflect the advantages of physics-informed learning (Section 2.2). The reviewed localization methods reveal tension between data-driven and model-based strategies. Deep CNN/RNN architectures provide powerful feature extraction, achieving sub-centimeter errors in laboratory plates, but their reliance on dense sensor networks and large training datasets poses challenges for on-aircraft deployment. FEM–ML hybrids, by contrast, achieve comparable localization with fewer experimental data and offer interpretable outputs consistent with structural mechanics, though they are vulnerable to inaccuracies in the numerical model. Aerospace certification contexts therefore face a dilemma: balancing the efficiency of black-box deep learning with the interpretability and physics consistency of model-based methods. Future solutions may emerge from hybrid physics-informed deep learning, which combines both strengths.

#### 4.2.3. Classification and Quantification

After damage localization, the next step is to classify and quantify it. These correspond to levels three and four of the typical SHM process. In the following, all the articles that study damage classification and quantification are presented (see Table 7).

The study [46] focuses on damage classification in carbon/epoxy composite specimens by using acoustic emissions. AE signals are acquired during three points bending tests conducted on three specimens, containing no damage, cracks, and delamination. After applying PCA to reduce the amount of data, the K-Means algorithm and the SVM classification model are used to derive the type of damage, namely matrix cracking, delamination, fiber/matrix debonding, and fiber breakage. The results reveal that the two algorithms present similar accuracy in most cases.

Acoustic emissions, the K-Means clustering algorithm, and digital image correlation (DIC) are used in [47] to characterize the intra-/interlaminar damage modes of CFRP composite laminates. Tensile tests are used to study interlaminar damage, while mode I, mode II, and mixed-mode tests are used to analyze intralaminar damage, such as delamination. The K-Means algorithm enables distinguishing the different damage modes by clustering the AE extracted data. By means of DIC, it is possible to analyze both the displacements and strains, to assign a specific damage mode, i.e., fiber breaking, matrix failure, debonding, and delamination, to one of the clusters. This method is therefore suitable for the SHM of composite laminates.

Lamb waves and deep neural networks are used by Huang et al. [67] for defect detection and classification in CFRP composite plates. Different features are extracted from the signals for three different plates, namely intact plate, hole plate, and crack plate. The authors employ different algorithms for generating and improving the quality of data, such as the Generative Adversary Network (GAN) and Denoising Diffusion Probabilistic Model (DDPM). Then, the residual deep neural network DenseNet is trained to classify the damage-related features. Different algorithm combinations are tested on experimental data, and among them, the most promising one proves to be the DDPM + DenseNet combination, which, with a high accuracy, enables the detection and classification of damage in the CFRP composite plate under examination.

Berghout et al. [68] introduced a biologically inspired CNN with reversed mapping (CNN-RM) to improve SHM interpretability in military training aircraft. By introducing feedback connections, their model achieved higher testing accuracy (95.1%) compared to standard CNNs (94.2%) on vibration-based datasets. More importantly, the feedback mechanism provided a pathway toward explainable ML decisions, directly addressing the certification bottleneck for aerospace deployment.

In the study [146], ultrasound and ML algorithms are used for damage diagnosis, involving both damage localization and quantification, in a composite CFRP panel. To experimentally simulate different damage conditions, a mass is positioned on the structure at various points. After signal collection and pre-processing, features are extracted to implement different supervised classification algorithms. These are tested for both localization and quantification tasks, and the results demonstrate that for locating the damage, the higher accuracy is reached by the Bagged Trees algorithm (that belongs to the decision trees family), while for the damage size estimation, the Ensemble-Subspace KNN algorithm reaches the higher accuracy value.

The authors in [147] utilize the SVM algorithm for damage localization and quantification. After collecting data from Lamb waves propagated on a glass/epoxy composite plate, on which different metal blocks are positioned to simulate different intensity delamination, an SVM algorithm is employed to derive the damage position. Then, the Fisher clustering method is used to derive the optimal detection path from which features can be extracted to train another SVM classifier to eventually classify the damage (in terms of severity). The authors conclude that the method can be applied to composite plates using only a few PZT transducers, obtaining high accuracy in both multi-area damage localization and quantification.

Zeng et al. propose two studies [148,149] for circular damage localization and quantification in CFRP composite panels, utilizing Lamb waves. In [148], the authors acquire Lamb-wave data of the pristine structure, and then they drill holes to simulate damage to train the imaging algorithm based on the Continuous Hidden Markov Model (CHMM). The algorithm derives the possible damage location and its related features. The damage size is then derived from a mathematical expression. In [149], the same authors use a combination of Levenberg–Marquardt (LM) and Quantum-Inspired Gravitational Search (QGSA) algorithms for damage localization, analyzing the Lamb-wave scattering sources. Then, the circular damage dimension is derived by drilling a CFRP panel and using non-linear equations solving algorithms. The results are satisfactory for both studies. Furthermore, the implementation of the proposed methods is simple and intuitive, making them easily adaptable to online monitoring of composite structures, with the only limitation being the nature of the method, which considers only circular damage.

In the study [150], the NASA Ames Prognostics Data Repository is used to acquire Lamb-wave data for three different CFRP composite layups, which refers to different specimens presenting different damage states. To conduct the analysis, the authors refer to only one element of the database. Statistical features are extracted in both time and frequency domains. Then, four different algorithms—KNN, SVM, DT, and RF—are trained in two steps: the first step consists of training the algorithm on a set of the database that contains the damage presence information, while the second step consists of training the algorithm on a set of the database containing damage position and type (crack or delamination) information. The results demonstrate that the DT algorithm reaches better performance when features are extracted in the time domain, while the RF algorithm is better if features are extracted in the frequency domain.

The authors of [151] utilize supervised and unsupervised algorithms to classify damage in GFRP composites under fatigue loads. The supervised linear regression and multiple linear regression algorithms are employed to find a relationship between AE parameters and damage-sensitive parameters, namely type and severity of the damage. The K-Means algorithm is used to cluster AE data based on the type of damage in an unsupervised context, i.e., matrix cracking, fiber pullout, and fiber debonding. All the analyzed models prove to be accurate in predicting the type of damage, starting with both labeled and unlabeled data.

Duan et al. [152] present a methodology to detect and visualize both the location and size of fatigue damage in CFRP composite laminates. Lamb-wave signals are collected via PZT sensors, and by means of a Bayesian learning algorithm, they are reconstructed through sparse representation. The output of this algorithm is the damage factor evaluated from the peak value and the peak time variation between the healthy signal and the damaged signal. This damage factor is subsequently used as input for a probability imaging algorithm that allows visualization of the damage as an image. The images obtained are consistent with the location and size of real fatigue damage, demonstrating the good performance of the proposed method.

The following studies utilize a model-based or hybrid approach, unlike the previous ones. Pagani et al. [153] use a vibration-based model and an ANN to predict the position and severity of damage in composite structures. Monte Carlo simulations enable the generation of a damage-based dataset. Then, a free-vibration analysis is carried out to extract the natural frequencies, in combination with the Modal Assurance Criterion (MAC) scalars, to train the ANN model. The network correctly predicts damage position and severity after its validation via a composite plate model.

Shirazi et al. [154] use a similar approach. After modeling a composite plate, the authors use an ANN trained on vibrational data to classify damage, modeled as a percentage reduction in stiffness. The first step, namely damage localization, is carried out by evaluating the modal strain energy change ratio (MSEcr), and subsequently, the ANN, in combination with the YUKI optimization algorithm, allows prediction of the damage level.

The authors of [155] analyze damage classification and quantification, i.e., damage type and severity. In this work, nonlinear model-based features (NMBFs) and signal-based features (SBFs) are derived either from numerical data (composite beam model) or experimental data (CFRP composite plate). After utilizing PCA for data size reduction, an SVM algorithm is trained on different scenarios: (i) only SBF for training, (ii) only NMBF for training, (iii) PCA on SBF and NMBF with only two principal components for training, and (iv) PCA on SBF and NMBF with only three principal components for training. The results show that by using NMBF, SVM performance improves both damage classification and quantification. Moreover, the PCA algorithm improves classification performance.

A Bayesian framework for damage detection, classification, and quantification is employed in [156] for CFRP composite beams. The authors analyze two numerical cases, which are distinguished by damage type and delamination. FEM simulations generate numerical data for each damage type by subjecting the examined structures to ultrasonic guided waves. First, a probabilistic mathematical model of the structure is created, on which Bayes theorem is applied in two levels: the first to understand damage position, the second for damage type. The model is updated starting from ultrasonic signal data to detect the damage position, and then, the Metropolis-Hastings algorithm allows us to derive the damage type in the form of probability. The proposed method has the advantage of working on raw data, rather than extracted features.

This body of work illustrates how both ensemble methods and dimensionality reduction (Section 2.2) improve classification performance, while recent deep generative models (Section 2.2) support data augmentation and robustness in defect classification. Classification studies demonstrate that ensemble methods and dimensionality reduction techniques can improve robustness to noise and high dimensionality, while deep generative models (e.g., GANs, DDPM) enhance data augmentation and defect differentiation. However, performance variations often reflect the sensitivity of different algorithms to feature design: decision tree ensembles depend on carefully engineered statistical features, while deep CNNs and autoencoders learn features directly but require large, balanced datasets. Aerospace SHM faces the additional challenge of translating classification accuracy into actionable maintenance decisions; a model that distinguishes delamination from fiber breakage with 98% accuracy is valuable only if it also reliably quantifies severity. Thus, the methodological trade-off here is between feature efficiency, interpretability, and operational utility.

### 4.3. Fatigue

Residual useful life of composite structures is strictly related to the concept of fatigue. This section presents various research articles referring to fatigue prognosis (see Table 8).

The authors in [48] estimate the fatigue life of GFRP composites by means of AE data. Four algorithms are employed to quantify damage, three of which are ensemble learning algorithms, namely XGBoost, LightGBM, and CatBoost, and one an unsupervised K-Means clustering algorithm. Sixteen GFRP specimens are subjected to fatigue loads, and simultaneously, AE signal data are collected for the subsequent features extraction phase. The three ensemble algorithms are used to predict AE parameters and rank features, comparing them to the real measurements that are strictly related to different damage mechanisms. The results show an agreement between predicted and actual damage types, and the XGBoost algorithm proves to be the best one. Ensemble learning algorithm predictions are Shapley additive explanation (SHAP) values that identify the most relevant AE features. The K-Means algorithm is employed to cluster signals starting from SHAP values and AE parameters, but it appears to be less reliable than the other algorithms, since finding the optimal cluster number is not trivial.

Structures subjected to fatigue loads experience a reduction in residual useful life (RUL), which is evaluated starting from the health indexes (HIs). For this reason, Moradi et al. [69] developed a method for HI evaluation by means of a semi-supervised deep neural network (SDNN) for a carbon/epoxy composite plate under fatigue loads. AE data in time and frequency domains are used as features in a semi-supervised context. Only the features that meet the three criteria of prognostic parameters, namely monotonicity (Mo), prognosability (Pr), and trendability (Tr), can be used as HI. A Bayesian optimization method is employed to identify the optimal hyperparameters for the SDNN and the leave-one-out cross validation for validating the model. By testing the model on the experimental data, the results show that the extracted HIs meet the three criteria.

In [70], AE data coming from impact and compression–compression fatigue tests on composite panels are utilized to extract statistical features for the subsequent development of two models for RUL prediction. After applying PCA to AE data (only 10 principal components), a time-independent model (TIM) is developed by means of a multilayer perceptron (MLP) algorithm that is optimized by Bayesian optimization (BO). The TIM allows evaluation of the HI of the structure in a time-independent way, called “1st level HI”. Then, via an LSTM algorithm, a time-dependent model (TDM) is developed, allowing the evaluation of the so-called “2nd level HI” after a time-based resampling of the previously calculated HI. RUL evaluation is obtained by meeting the three prognostic criteria, namely Mo, Pr, and Tr. The results demonstrate that RUL estimation is improved by considering the TDM for HI evaluation.

Galanopoulos et al. [157] take one step further than the previous study by evaluating RUL. Three different compression–compression fatigue cases are considered for the experimental campaign, namely (i) constant amplitude fatigue, (ii) variable amplitude fatigue, and (iii) casual amplitude fatigue, on CFRP panels. FBG sensors permit obtaining strain data, utilized by a Genetic Algorithm (GA) to evaluate the HI of the structure. In that case, HI evaluation is based on two prognostic criteria, namely, Mo and Pr. Subsequently, via a Gaussian process regression (GPR), RUL can be evaluated. The main advantage of the proposed methodology is that it is material-independent and hence can be generalized to any SHM application.

In a similar way, in [158], constant amplitude fatigue and variable amplitude fatigue compression tests are considered on CFRP composite panels, under the investigation of AE, for RUL evaluation. Only the AE data-based features that meet the three prognostic parameters criteria are employed as HI and utilized for training two algorithms, namely Gaussian process regression (GPR) and bootstrapped neural network (BNN). These algorithms serve as RUL evaluators by means of two approaches: multivariate, employing 10 features, and single feature-based, employing the best feature. By means of a single feature-based approach, RUL estimation near the end of life (EoL) of the specimens is found to be less accurate, and the multivariate approach proves to be better. In addition, the GPR algorithm requires less training time than the BNN.

The same authors [157], based on the results obtained in that study, develop a technique for RUL estimation of multi-stiffened panels (MSPs) using single-stiffened panel (SSP) data [159]. The HI of SSPs is employed to train two algorithms, namely GPR and Long Short-Term Memory (LSTM) networks, to estimate MSPs’ RUL. An ensemble learning technique is used to make better predictions by combining the outputs of the two algorithms and finding dynamic similarities between the SSPs and the MSPs for the final RUL estimation. The results demonstrate that RUL estimation is close to the real one, especially near the EoL, and strongly depends on the HIs.

Galanopoulos et al. [160] propose different HIs obtained from AE, as well as strain data (utilizing FOSs), for stiffened carbon/epoxy composite panels subjected to two fatigue test campaigns. Eight HIs are evaluated: four strain-based HIs (HI1 to HI4), two virtual HIs (vHI1, and vHI2), and two AE-based HIs. PCA is used to extract principal components, which are used as virtual HIs. The results show that HI3, HI4, vHI1, and vHI2 display highly monotonic behaviors, meeting the Mo criterion, but all strain-based HIs have relatively poor prognosability. The two acoustic-based HIs present lower monotonicity, but higher prognosability, meeting the Pr criterion.

The authors of [161] carry out fatigue cycle tests on CFRP coupons until failure to predict the fatigue damage index. Lamb waves are employed to generate a database for the proposed algorithm, namely a Maximum Entropy (ME) algorithm, which is in the family of KNN algorithms. The performance of the ME algorithm is compared to other state-of-the-art algorithms, such as KNN, Gradient Boosting Trees (XGBoost), and neural networks. Coupons have a notch to cause delamination, and two six-PZT-SMART layers are embedded into them. The Palmgren–Miner index is the algorithm output used to estimate fatigue damage. The proposed algorithm proves to be better than the weighted-KNN (W-KNN). Moreover, it does not require training and hypertuning; thus, its computational time is lower than the other considered algorithms.

The authors of [162] propose a methodology for fatigue damage diagnosis, which includes damage detection and classification. A CFRP composite plate is first subjected to uniaxial tensile fatigue cycles (<2.5 thousand cycles), and Lamb-wave data are collected for training a deep autoencoder (DAE) algorithm. Then, hyperparameter optimization has improved accuracy and sensitivity. A statistical baseline that employs reconstruction errors is introduced for damage detection. Additional fatigue cycles (≥2.5 thousand cycles) are enforced on the plate, and based on the statistical baseline, matrix cracking and delamination can be identified. Clustering is done by a Density-Based Spatial Clustering of Applications with Noise (DBSCAN) algorithm, which classifies data into three classes: intact, matrix cracking, and delamination. The DBSCAN algorithm clusters data by analyzing dominant features (automatically extracted by DAE) that are dimensionally reduced by the single value decomposition (SVD) method. The main advantage of this method is that it does not require manual feature extraction, so computational times are reduced.

A K-Means++ algorithm is developed in [163] for fatigue damage mode clustering in CFRP open-hole laminated beams under constant amplitude tensile–tensile fatigue loads. AE and DIC are the two techniques employed. Firstly, eight damage-correlated characteristics are collected from AE data, which are then processed through Laplacian score (LAS) to obtain only four relevant features. Then, PCA is employed for extracting the principal components from the features. The algorithm allows for clustering data such as delamination, fiber breakage, matrix cracking, and fiber pull-out. DIC permits evaluation of the strain state of the specimens’ surface. AE and DIC data are used to evaluate the damage evolution through the cumulative damage index (CDI), which is defined as the ratio between the cumulative number of AE events of different damage modes and the cumulative number of AE events. The results show that the AE technique can predict fatigue failure in real time, compared to DIC.

The use of RNNs and LSTMs for fatigue prognosis directly echoes the discussion in Section 2.2, confirming their strength in modeling temporal dependencies, while Gaussian process regression (Section 2.2) provides interpretable probabilistic predictions valuable in aerospace certification contexts. Fatigue prognosis highlights perhaps the starkest methodological contrasts. Gaussian process regression and Bayesian methods provide interpretable, probabilistic RUL estimates, crucial for certification and risk management, but they struggle to scale to large datasets. Neural networks and recurrent models (LSTM, GRU) effectively capture long-term temporal dependencies and degradation trends, but risk overfitting and lack transparent uncertainty bounds. Health indicator (HI) construction further drives performance differences: while monotonicity and prognosability criteria yield reliable predictors in controlled tests, their robustness under variable flight loading is less clear. Aerospace deployment, therefore, requires hybrid approaches that combine the interpretability of probabilistic models with the temporal expressiveness of deep networks, ensuring both predictive accuracy and certifiable reliability.

### 4.4. Impacts

This section covers research articles concerning impact damage (see Table 9).

Ai et al. present a study [164] regarding automatic impact detection and localization, employing two algorithms, namely Random Forest and Stacked Autoencoder (SAE). The experiment consists in dropping a steel ball on a composite aircraft elevator, such that AE signals can be collected. The RF algorithm is trained on a dataset of 3600 AE samples to find the source localization by using 15 features, and the labels represent the zone number of the impact. The SAE is trained on both AE raw signals and their fast Fourier transform (FFT), and the output is the impact localization. The results obtained are compared with a traditional ANN and show that the accuracies for RF, SAE, and ANN, are, respectively, 98.3%, 99.2%, and 96.0%. SAE proves to be the most accurate algorithm but requires higher computational time and input storage than the other algorithms. Considering that a large amount of AE data (related to impacts) could be collected during flight, the RF algorithm proves to be better for both computational time and input storage.

The same authors, in [165], utilize deep neural networks to localize impacts on a real-sized aircraft elevator. The experimental tests involve dropping a steel ball on both ribs and panel (area between ribs), resulting in a total of 4800 impacts. AE data are collected to train SAE networks. The first network classifies the impact zone as either “panel” or “rib”, while the second network localizes the impact zone. The accuracy of the first network is 98%, while the accuracy for the zone localization network is 99.2%. The results indicate that employing frequency domain AE data instead of time domain data improves the accuracy of the networks. Furthermore, the proposed framework is also tested by increasing the number of localization zones to detect, from three to twenty, demonstrating that the SAE network is also efficient in that scenario.

Because of the uncertainty of impact location, angle, and energy, the authors of [166] use a Bayesian neural network (BNN), a single-ANN, and a muti-ANN for classifying energy levels and quantifying this uncertainty. A composite flat plate, with PZT passive sensors, is used for the impact test campaign, consisting of impacts with different levels of energy and different angles. The findings exhibit that both BNN and single-ANN can classify energy levels of perpendicular impacts with high accuracy and the uncertainty for perpendicular impacts, but computational costs of the multi-ANN are larger than the BNN. However, for angled impacts, both the BNN and the multi-ANN previsions cannot reach an accuracy higher than 50%. To conclude, the developed algorithms can classify the energy levels and quantify the uncertainty for the perpendicular impacts, but show low performance for the classification task of angled impacts.

In the study [167], localization of low-velocity impacts (LVIs) is done by means of a genetic algorithm. Firstly, recurrence quantification analysis (RQA) is performed by using data from a recurrence plot. Recurrence Rate (RR) and Determinism (DET) are used for investigating the response signals of LVI. Then, a genetic algorithm is employed to predict impact location by utilizing estimated distances between impact and FBG sensors, obtained by RQA. The experimental results, conducted on a composite 500 × 500 mm plate with 6 FBG sensors, show that the proposed algorithm is efficient, and the average localization error is 25.27 mm.

The authors in [168] propose a method for autonomous BVID recognition, employing four different DL algorithms, namely ConvNet, ResNet, a prototypical network based on ConvNet, and a prototypical network based on ResNet, all belonging to the CNN family. Surface images of different composite samples are used as input for the algorithms’ training phase. Different impact tests are performed by varying the impact energy. Each algorithm is tested on the following tasks: (i) detect damage on the back face of the samples, without employing sensors; (ii) detect damage on the front face of the samples, without employing sensors; (iii) detect damage on the back face of the samples, with an embedded glass/carbon composite sensor; (iv) detect damage on the front face of the samples, with an embedded glass/carbon composite sensor. ResNet proves to be the best model in performing all four tasks, and, moreover, performance increases by embedding a sensor within the structure.

In the study [169], CNNs are used for impact localization and characterization in composite plates. Impacts on a composite stiffened curved panel are recorded via PZT sensors by varying the dropping height from 20 mm to 80 mm, with steps of 20 mm, to obtain different energy levels. For training the “location prediction” CNN, 2D images are obtained from the recorded signals by selecting the sensors closer to the impact event, considering signals amplitude. The next step is to predict the energy level of the impact and to characterize the impact into three labels, namely “Safe”, “Alert”, and “Danger”. Two methods are employed to generate 2D images for training the “energy prediction” CNN. These methods consider the area under the absolute value of the signal amplitude and the averaged storage energy for each sensor, respectively. The method accuracy is between 94.3% and 10% in predicting the impact location, while it is over 98.3% in classifying impacts energy. The proposed model is then tested on impacts for which the position is different from that in the trained ones and shows an accuracy of over 95%. Moreover, the developed CNNs do not require a huge amount of data for training.

As in the previous article, Damm et al. [170] also propose a method for impact localization and energy characterization in CFRP plates, using embedded microelectromechanical system (MEMS) sensors. The study involves two plates, one with embedded MEMS sensors and the other with PZT sensors. Two CNNs are trained on spectrograms extracted from the acquired signals. A comparison between the use of PZT and MEMS sensors is conducted, revealing that the CNNs trained on MEMS data achieve accuracies of 99.76% and 97.04% for localization and classification, respectively. The CNNs trained on PZT data exhibit accuracies of 99.58% and 98.68%.

Smart composite samples are manufactured and employed by the authors of [171] to obtain impact data, which is converted into images by discrete wavelet transform (DWT), for the subsequent training of a CNN algorithm to perform impact characterization. LVI impact tests on the composite samples are carried out at predetermined locations. A Bayesian optimization algorithm is used to tune CNN hyperparameters, achieving efficient impact localization with an average error of 13.8%. A second CNN characterizes failures under three LVI energy levels at the specimen center, using time–frequency analysis to identify failure modes, with an average error of 11.3%. Data augmentation, applied by shifting or altering image pixels, further reduces the error in both localization and classification tasks.

In [172], an impact localization algorithm is developed based on a Radial Basis Function (RBF) interpolation method. A CFRP composite 300 × 300 mm plate is employed to conduct impact tests at different energy levels (by varying the drop height) on specific points of the structure for the initial calibration. The data acquired via PZT sensors are used to train the algorithm. The exact location of the impact is evaluated by applying the Time Reversal (TR) method, i.e., correlating baseline signals to new impact signals. Then, via RBF interpolation, it is possible to train the algorithm.

Seno et al. [173] analyze two different types of impacts, namely “hard” and “soft”, simulating different kinds of impactor stiffness. Hard and soft impacts are introduced using steel and silicone heads, respectively, with PZT sensors recording the response on a composite plate. Impact localization relies on two features: time of arrival (ToA) and signal amplitude ratios. The ToA is extracted using either the Normalized Smooth Envelope Threshold (NSET) or a modified Akaike Information Criterion (AIC). A Database (DTB) method, similar to ANNs, compares input impact features to baseline signals and is benchmarked against a reference ANN. While DTB–AIC performs comparably to ANN–NSET for hard impacts, it proves superior for soft impacts.

The same authors, in [174], employing the same method, investigated the impact location on flat and curved composite plates under different environmental and operational conditions. The authors conducted the experimental tests by increasing temperature, increasing height, increasing mass, and utilizing angled impacts. The results showed a high accuracy of the ANN-NSET framework, and that it is not affected by the number of sensors and plate geometry.

Zhao et al. utilize a model-based approach to predict the Compression After Impact (CAI) strength of carbon/glass hybrid laminates [175]. The training database is created starting from an FEM of the structure, which permits obtaining both impact parameters, such as impact time and impact energy, and CAI strength. An XGBoost algorithm is trained on that database and subsequently tested on the experimental data, obtained by impact tests on the laminates. The residual compressive strengths of the laminates are evaluated via a compression test and compared with those predicted by the algorithm, resulting in only 3.55% error. Hence, the proposed XGBoost algorithm demonstrates its superiority over the FEM model in predicting CAI strength, for both prediction error and computational time.

Recent studies extend beyond traditional impact localization towards dual-task frameworks. For instance, Nicassio et al. [176] trained shallow neural networks to both regress impact energy and classify damaged zones in reinforced aluminum aerospace panels. Their approach achieved ≤10% error in energy estimation and >95% accuracy in zone classification, showing that physically meaningful feature selection combined with lightweight ML models can outperform deeper “black-box” architectures in terms of efficiency and interpretability. Similarly, Humer et al. [177] demonstrated end-to-end recurrent neural networks (GRUs) trained directly on Lamb-wave sensor data for impact localization on thin-walled structures. Importantly, their work used real-world experimental datasets, moving beyond purely numerical simulations, which represents a critical step towards operational readiness.

Impact detection and localization tasks clearly benefit from the pattern-recognition capabilities of CNNs and autoencoders (Section 2.2), while Bayesian methods remain attractive for quantifying uncertainty in impact energy estimation. Impact detection and localization methods reveal a clear divide between lightweight, interpretable models and high-capacity deep networks. Random Forests and Bayesian neural networks offer fast, uncertainty-aware classification suitable for real-time flight monitoring, but their accuracy lags slightly behind deep CNNs or SAE architectures trained on rich datasets. CNN-based methods achieve near-perfect localization in controlled tests, yet their generalization collapses under angled or low-energy impacts, where signal features overlap strongly with background noise. Performance variations are thus not simply algorithmic, but rooted in data representation, impact variability, and sensor deployment density. For aerospace practice, robustness to environmental and operational conditions remains a greater bottleneck than laboratory accuracy, underscoring the need for uncertainty quantification and adaptive, hybrid frameworks.

### 4.5. Others

The following section describes the research articles related to ML applications for SHM, including the study of cracks, the correct position and number of sensors, and other aspects of health monitoring (see Table 10).

Liu et al. [178] propose a Gaussian Process (GP) algorithm to detect crack damage in a Nomex honeycomb-core/CFRP-skin composite sandwich plate, with numerical data. Vibration responses of healthy and cracked sandwich plates, under different frequencies, are collected via numerical simulations. DWT is applied to the collected data before training the GP algorithm. The performance of the GP algorithm is investigated by using three sensors, followed by only one sensor. By employing all three sensors, the best results are obtained, while with only one sensor, an accuracy of 100% can be reached, but misclassification can arise.

In [179], crack length and orientation are predicted by two algorithms (polynomial regression algorithm and ANN). The electrical resistance change (ERC) technique allows us to obtain electric conductivity values for CFRP twill-woven plates, which are used to create an FEM model of the structure. Data obtained from the FEM model are employed by two algorithms to predict crack length and orientation, from a new electric conductivity value. Polynomial regression with Leave-One-Out Cross-Validation (LOOCV) proves to be better than ANN for the prediction task.

Crack length evaluation is performed by the authors of [180] with the Enhanced Jaya ANN (E-Jaya-ANN) algorithm. Modal analysis, through FEM simulation, is conducted on GFRP composite specimens with different crack lengths to extract natural frequencies. The FEM model is validated by experimental tests, i.e., bending, tensile, and modal analysis tests, resulting in an error lower than 1.25%. Natural frequencies are the input for the E-Jaya-ANN algorithm, which returns crack lengths as output. The algorithm with higher accuracy is the one with eight hidden neurons.

Since it is difficult to identify cracks in sandwich composites, images obtained via scanning electron microscopy (SEM) are utilized for training three different algorithms, namely SVM, KNN, and deep CNN (DCNN), in [181]. These algorithms allow classification of the glass fiber-reinforced PU foam cored epoxy/vinylester sandwich composite as damaged or undamaged. The features extracted from the SEM images are mean, standard deviation, entropy, variance, smoothness, kurtosis, and skewness. SVM, KNN, and DCNN reach an accuracy of 78.23%, 85.56%, and 94.33%, respectively.

The classification of crack location and severity in composite laminates are investigated by Shirazi et al. [182]. An FEM model of the GFRP composite beam enables modal analysis to be conducted. The model is then validated through experimental tests with hammer strikes. Vibration responses of the simulated hammer strikes are employed as input of the 1D-CNN algorithm. The convolutional layers of the CNN automatically extract features from the input data. Single damage and double damage are two analyzed scenarios. Subsets of vibration responses of single/double-damaged beams, with different crack locations and intensities, are used as training data. For the single damage classification scenario, network accuracy is 95%, while for the double damage classification scenario, accuracy is 93%. In addition, merged subsets of single and double damage data are used to train the algorithm, resulting in an accuracy of 89.6%. In this case, by optimizing the hyperparameters, accuracy improves to 92%.

Zhu et al. [183] develop an automatic crack classification algorithm based on the U-Net deep learning model in 3D woven composites. The algorithm can identify cracks in the warp, weft, resin, and interfacial cracks. The algorithm is trained on computed tomography (CT) images to distinguish warp and weft. Then, the CT image segmentations are derived under three impact pressures and based on the segmentation results: the algorithm automatically classifies the different cracks, with an accuracy of more than 88%. The main advantage of this model is that compared to manual classification, computational times and efficiency are improved.

Matrix cracking classification is investigated by the authors of [184], utilizing three different algorithms, including SVM, and two neural networks, namely linear vector quantization (LVQ) and MLP. Ten cross-ply glass/epoxy laminated composite specimens are subjected to uniaxial tensile tests to induce matrix cracking with different densities. Lamb-wave propagation through each specimen allows us to extract three features: wave velocity, wave amplitude at four actuator–sensor distances, and three ratios of the wave amplitudes. These features are used to train the three algorithms prior to a linear discriminant analysis (LDA) to find a linear combination of features for a subsequent better classification. The results show that the SVM and LVQ outperformed the MLP, with the SVM having the highest accuracy of 91.7%, which can improve to 96%, by considering only three classes (intact specimens and two level of matrix cracking specimens) instead of four (intact specimens and three level of matrix cracking specimens).

Zhang et al. [185] study the mechanical deformation, strength, and progressive damage behaviors of a CFRP laminate with circular holes. They employ an FEM model to conduct a laminate failure analysis, generating a dataset of the stress-strain state and the strain increments. This dataset is used to train the ANN, which predicts the mechanical behavior of the laminate under different load conditions. The results exhibit that the proposed ANN can predict the mechanical deformation, strength, and progressive damage in laminates with circular holes, offering a time reduction in the global monitoring of structures.

The objective of the study [186] is to develop predictive models for bending and torsional load spectra on three different sections of an aircraft wing under both buffet and maneuver loads, represented as load cycles for subsequent fatigue life evaluation. The authors investigate the following algorithms: (i) auto-regressive models, such as Auto-Regressive with eXogeneous Input Model (ARX) and Auto-Regressive Moving Average with eXogeneous Input Model (ARMAX); (ii) artificial neural networks, such as Cascade Forward (CFN), Time Delay (TDN), Layer Recurrent (LRN), and Nonlinear Auto-Regressive (NXN) networks; (iii) recurrent neural networks (RNNs), such as LSTM and bi-directional LSTM (Bi-LSTM). The dataset is generated from defense fighter air platform flight data, collected by strain and loading wing sensors. The analysis is divided into maneuver loads and buffet loads, with each algorithm applied to bending or torsion loads. Bending loads at the root and mid-span can be predicted by auto-regressive models and ANNs, while torsional loads require the employment of RNNs. The results indicate that ANNs prove to be superior compared to ARX and ARMAX, while the only model that can predict tip torsional loads is the Bi-LSTM.

ML algorithms can be applied to other applications, such as monitoring the manufacturing phase or choosing the correct number and orientation of sensors for SHM.

The study [187] aims to develop an ML algorithm for real-time monitoring of natural fiber-reinforced plastic (NFRP) composites. In particular, the purpose of the study is to monitor changes in cutting speed and fiber orientation during machining. Experimental tests are conducted to extract the spectral features of AE signals. An RF model is then trained on these features to distinguish “cutting” and “non-cutting” phases and, additionally, to predict the fiber orientation. The proposed method is then compared to Fisher’s linear discriminant analysis, and the RF model demonstrates superior performance, achieving an accuracy of 94% in classifying “cutting” and “non-cutting”. The same algorithm has an accuracy of 95% in distinguishing fiber orientations toward the cutting direction.

An et al. [188] propose a methodology to optimize the number and orientation of sensors for two case studies, i.e., a “clamped-clamped composite plate” and a “composite stiffened cantilever panel”. The main purpose is to enhance the efficiency of vibration-based damage detection by optimizing the sensor network. The Modal Assurance Criterion (MAC) is employed as a metric to assess the number of sensors, while the Modal Kinetic Energy (MKE) index is employed to determine the optimal sensor positions. The Nondominated Sorting Genetic Algorithm II (NSGA-II) explores the possible sensor layouts, and the Monte Carlo simulation (MCS) evaluates the values of MAC RMS errors. A GPR model is employed to reduce computational costs. An FEM model provides the dynamic responses of the structure, validating the NSGA-II model. The whole framework is applied to two case studies, implemented via the FEM, resulting in the optimization of sensor layout. Eventually, the framework is used to detect delamination, as an inverse problem, thereby validating the optimized sensor placement.

Neural networks have also been applied to acoustic source localization. In [189], a backpropagation (BP) network was trained on both numerical and experimental datasets, generated, respectively, from anisotropic structure modeling and PZT/SLDV measurements, to localize a five-peak narrow-band sinusoidal excitation. The BP network achieved 100% accuracy on the numerical dataset, with slightly lower performance on experimental data due to environmental variability.

Sikdar et al. [190] proposed a CNN-based method for breathing-debond assessment in stiffened composite panels (SCPs). Ultrasonic signals from a PZT network were used to validate FEM models and generate training data. CNNs trained on scalograms (via CWT) classified debonds with 85.6% accuracy, which improved to 95.9% when trained on higher harmonic (HH) signals extracted from the raw data.

These additional applications further demonstrate the flexibility of the ML algorithms introduced in Section 2, from regression models for crack length prediction to optimization-guided ANNs for structural debonding assessment.

## 5. Critical Synthesis and Adoption Challenges of ML for Aerospace SHM

The preceding sections have reviewed a wide range of machine learning methods applied to the main structural health monitoring (SHM) tasks—damage detection, localization, classification/quantification, and fatigue prognosis. While individual studies report high accuracies, direct comparison is challenging due to differences in experimental setups, sensor networks, environmental conditions, and data availability. To distill the key findings and guide future development, Table 11 summarizes the suitability of different ML approaches for each SHM task, highlighting their main strengths, limitations, and indicative technology readiness levels (TRLs).

From the table, the following observations can be formulated:

- Most mature approaches (TRL 5–6) are supervised learning- and hybrid model-based methods for detection and localization under controlled conditions. These are close to operational readiness but require more flight validation.
- Emerging but less mature (TRL 2–4) are digital twin integration, federated learning, and physics-informed deep learning. While promising for operational robustness and adaptability, they remain at early experimental stages.
- Common barriers across methods include dataset scarcity and lack of standardized benchmarks, sensitivity to environmental variability (temperature, humidity, vibration), interpretability and certification challenges for “black-box” models, and real-time processing constraints for onboard SHM.
- Enabling technologies for advancing TRL include synthetic data generation (GANs, physics-based simulators), transfer learning to leverage cross-platform knowledge, adaptive algorithms capable of recalibration under changing conditions, and multi-sensor fusion for complementary damage indicators.

Building on this comparative assessment, several future directions can be identified. The scarcity of labeled flight data and the limited generalizability of lab-trained models remain the primary bottlenecks [191,192].

Improving robustness to operational variability—through adversarial training strategies [193], hybrid and physics-informed models, or federated learning—will be essential if ML is to be trusted in aerospace certification. For instance, recent studies suggest that federated learning can reduce centralized data requirements by up to 40–60% in fleet-wide SHM scenarios while preserving model accuracy [194,195], thereby alleviating data scarcity and privacy concerns across geographically distributed aircraft.

Equally important is addressing the interpretability challenge of “black-box” models, which is particularly critical for aerospace certification. Recent work has demonstrated the use of explainable AI (XAI) techniques, including SHAP and LIME, to attribute feature importance in vibration- and wave-based SHM [196,197], while attention mechanisms and saliency maps have been applied to highlight damage-relevant regions in structural images and spectrograms [198]. These methods provide transparent diagnostic cues that can be cross-validated with engineering knowledge, mitigating the opacity of deep learning. Eventually, the integration of XAI into digital twin frameworks supports lifecycle monitoring and continuous model adaptation, bridging the gap between predictive accuracy and operational trustworthiness.

Advances in embedded and wireless sensors, such as fiber optics, nanomaterials, and CNT networks [115], point towards lightweight, multifunctional monitoring solutions.

Ultimately, adoption will depend not only on algorithmic accuracy but also on regulatory alignment and cost-effectiveness. Certification bodies require interpretable, transparent models, and operators need assurance of scalability across fleets. As Kosova et al. [199] stress, integration with maintenance protocols and digital fleet management will be decisive. The next generation of SHM frameworks is therefore expected to evolve into adaptive, intelligent, and certifiable systems capable of autonomous damage diagnosis, real-time updates, and seamless digital-twin integration.

The adoption of ML-based SHM in aerospace structures is both a technical and regulatory challenge. In a safety-critical context, methods must comply with stringent certification expectations from the EASA and FAA, whose current frameworks were largely conceived for deterministic systems rather than data-driven models [116,117,118,119,120,121,122,123,124,125,126,127,128,129,130,131,132,133,134,135,136,137,138,139,140,141,142,143,144,145,146,147,148,149,150,151,152,153,154,155,156,157,158,159,160,161,162,163,164,165,166,167,168,169,170,171,172,173,174,175,176,177,178,179,180,181,182,183,184,185,186,187,188,189,190,191,192,193,194,195,196,197,198,199,200,201,202]. This creates hurdles for approval, particularly around lifecycle assurance of trained models, dataset representativeness, and in-service monitoring of model performance drift.

A central barrier is explainability and verification. High-capacity black-box models can deliver excellent laboratory accuracy, yet remain difficult to validate under certification scrutiny. Regulators expect evidence of robustness, traceability, and quantified uncertainty, making model interpretability, confidence measures, and explicit uncertainty quantification (UQ) key enablers for industrial uptake [200,201,202,203]. Data issues compound the problem: representative, labeled datasets across the operational envelope are limited, and confidentiality constraints restrict cross-program sharing.

From an industrial standpoint, adoption is slowed by integration costs (retrofit of sensor networks, data pipelines, and digital-twin interfaces), organizational conservatism, and the need to align ML workflows with existing airworthiness processes. Hybrid approaches—which fuse physics-based models with ML—are promising because they retain traceability and physical interpretability while leveraging data-driven performance. Progress will depend on codified assurance guidance (for lower-risk ML levels first), standardized datasets/protocols, and early engagement among OEMs, operators, and regulators [200,201,202].

## 6. Conclusions

Machine learning has emerged as a transformative tool for enhancing the performance and intelligence of structural health monitoring systems in aerospace engineering. This review has shown that ML enables early damage detection, localization, classification, and prognosis by leveraging data from advanced sensor networks.

However, the full potential of ML-based SHM is still constrained by challenges such as data availability, model interpretability, and the integration of heterogeneous sensor types. Continued research is needed to address these gaps and ensure the reliable deployment of such systems in real-world aerospace environments.

Future progress will likely hinge on the development of hybrid models combining data-driven and physics-based approaches, improvements in sensor technology, and the adoption of emerging paradigms such as transfer learning and digital twins.

In conclusion, the convergence of ML and SHM holds great promise for the aerospace sector. It represents a critical step toward achieving autonomous, intelligent, and adaptive structures capable of self-diagnosis and informed decision-making throughout their service life.

This review has deliberately followed a dual structure: first introducing the main families of machine learning algorithms in a concise yet rigorous way, and then critically mapping them onto structural health monitoring tasks for aerospace structures, thereby bridging methodological development with domain-specific applications.

By serving as a bridge between machine learning methods and aerospace SHM applications, this review provides both a reference framework for engineers seeking to apply ML in practice and a roadmap for ML researchers to address the unique challenges of aerospace structures.

## Figures and Tables

**Table 1:** Supervised and unsupervised machine learning methods in aerospace SHM applications.

Method	Typical Aerospace SHM Application	Strengths	Limitations
Support Vector Machines (SVMs)	Damage detection in composite plates using Lamb-wave features	High accuracy with small datasets; effective for binary classification	Requires labeled data; performance drops with noise or nonlinear damage states
Random Forests (RFs)	Impact energy classification in composite coupons; strain-based crack detection	Robust to noise; interpretable feature importance; fast inference (onboard suitability)	Less effective at learning complex feature hierarchies
k-Nearest Neighbors (k-NN)	Localization of low-velocity impacts in CFRP panels	Simple, no training phase; intuitive	Sensitive to feature scaling; computationally heavy for large datasets
Principal Component Analysis (PCA)	Novelty detection under varying flight loads and temperatures	No labels required; effective for dimensionality reduction	Sensitive to environmental and operational variability (EOCs)
Self-Organizing Maps (SOMs)	Clustering of acoustic emission data for damage characterization	Captures nonlinear structures in high-dimensional data	Interpretation not straightforward; prone to false alarms
Autoencoders (Unsupervised DL)	Detection of delamination in CFRPs without labelled data	Learns compact, task-specific latent representations	Higher false positives; requires large healthy-condition datasets

**Table 2:** Artificial neural networks (ANNs) and deep learning methods in aerospace SHM applications.

Architecture	Typical Aerospace SHM Case	Strengths	Limitations	Training Cost	Inference Cost
Multilayer Perceptron (MLP)	Strain-based damage detection in stiffened composite panels	Simple and interpretable; effective on small datasets	Limited feature extraction capacity; prone to overfitting	Low Scales with a number of layers/neurons; efficient on vibration/guided-wave features.	Very low Feasible on embedded devices (FPGA/MCU)
Convolutional Neural Networks (CNNs)	Impact localization using acoustic emission or Lamb-wave imaging	Learns spatial features; excellent accuracy in impact and imaging tasks	Needs large datasets; computationally demanding	Medium–High Cost grows with kernel size, channels, and input resolution; GPU recommended.	Medium Can be optimized via pruning/quantization
Recurrent Neural Networks (RNNs)/LSTMs/GRUs	Fatigue life prediction and remaining useful life (RUL) estimation	Captures temporal dependencies; effective for sequence modeling	Training instabilities; requires long time-series datasets	High Sequential training; scales with sequence length × hidden size	Medium–High Depends on input sequence length
Graph Neural Networks (GNNs)	Sensor network data fusion in distributed SHM of large airframes	Naturally handles graph-structured sensor layouts	Still emerging; limited aerospace datasets available	High Scales with a number of nodes/edges; computationally demanding for large structures.	Medium–High Inference costly if graph updates in real time
Generative Adversarial Networks (GANs)/Diffusion Models (DDPMs)	Data augmentation for rare impact or defect classes in composites	Expands dataset diversity; mitigates class imbalance	May generate unrealistic data; training complexity	Very high Adversarial training (generator + discriminator) requires long, unstable convergence	Medium Single pass at inference
Autoencoders/Variational Autoencoders (VAEs)	Unsupervised delamination detection; feature learning from guided-wave signals	Learns compact feature embeddings; effective for novelty detection	Sensitive to sensor noise; may reconstruct damage as “healthy”	Medium Similar to MLP/CNN depending on encoder complexity	Low Fast reconstruction suitable for real-time novelty detection

**Table 4:** Summary of damage detection research articles.

Article	Approach	Scope	Algorithms	Results
[26]	Data-driven	Damage detection on a composite sandwich wing’s front spar	SOM PCA	Accuracy of 98.1%
[27]	Data-driven	Damage detection in a carbon/epoxy plate	ANN	High resolution and generalization to any structure
[28]	Data-driven	Damage detection in a composite panel	Bayesian optimization algorithm	Reduction of time and computational costs
[29]	Model-based	Damage detection in a composite sandwich aeronautical spoiler	MLP	Numerical and experimental results demonstrate high efficiency
[137]	Model-based	Damage detection in composite laminates	ANN Metaheuristic optimization algorithm	Higher accuracy with respect to traditional ANNs

**Table 5:** Summary of damage localization research articles.

Article	Approach	Scope	Algorithms	Results
[65]	Data-driven	Damage localization in a CFRP composite plate	PAA GAF CNN	Localization error of 7.58%
[138]	Data-driven	Damage localization in a composite panel	1D-CNN gMLP	Small localization error
[139]	Data-driven	Damage localization in a composite sandwich	MDPI	High accuracy in detecting and visualizing damage position
[140]	Data-driven	Damage localization for complex composite structures	Imaging algorithm	Minimum localization error
[141]	Data-driven	Damage localization in a CFRP composite plate	DCNN recurrent regression algorithm	High accuracy and resolution. Only a few required sensors.

**Table 6:** Summary of damage identification and localization (combined) research articles.

Article	Approach	Scope	Algorithms	Results
[30]	Model-based	Damage detection and localization in a composite sandwich aeronautical spoiler	RF	High performance on numerical data; slightly weaker on experimental validation
[45]	Data-driven	Damage detection and localization in a CFRP composite panel	CAE TL	The method is scalable to any material
[66]	Data-driven	Damage detection and localization in a CFRP composite panel	CNN	Accuracies of 99.1% (detection) and 99.3% (localization) for damages
[142]	Data-driven	Damage detection and localization in a CFRP composite specimens	Imaging algorithm	Multipath Lamb waves reduce sensor requirements while maintaining accuracy
[143]	Model-based	Damage detection and localization in CFRP pin-joined truss structures	Hierarchical CNN	Model generalizes well compared with non-hierarchical approaches
[144]	Model-based	Damage detection and localization in a carbon/epoxy laminate	1D-CNN 2D-CNN CNN LSTM	2D-CNN for damage detection. CNN and LSTM for damage localization
[145]	Model-based	Damage detection and localization in the Airbus A350 composite wing	FEM + CNN	Accuracy of 99% in damage detection

**Table 7:** Summary of damage classification and quantification research articles.

Article	Approach	Scope	Algorithms	Results
[46]	Data-driven	Damage classification in carbon/epoxy composite specimens	PCA K-Means SVM	Similar accuracy in detecting the type of damage
[47]	Data-driven	Damage classification in CFRP composite laminates	K-Means	The algorithm distinguishes the different damage modes
[67]	Data-driven	Damage detection and classification in CFRP composite plates	GAN DDPM DenseNet	Highest accuracy for the DDPM+DenseNet combination
[68]	Data-driven	Component-level SHM in military trainer	CNN-RM	Higher testing accuracy by introducing feedback connections
[146]	Data-driven	Damage localization and quantification in a CFRP composite panel	Supervised classification algorithms	Bagged Trees, with the highest accuracy for localization KNN, with the highest accuracy for quantification
[147]	Data-driven	Damage localization and quantification in a glass/epoxy composite plate	SVM	High accuracy with only a few PZT transducers
[148]	Data-driven	Damage localization and quantification in a CFRP composite panel	Imaging algorithm	Simple and intuitive proposed methods easily adaptable to online monitoring of composite structures
[149]	Data-driven	Damage localization and quantification in a CFRP composite panel	CHMM + quantitative imaging	2D-CNN for damage detection; CNN and LSTM for damage localization
[150]	Data-driven	Damage localization and quantification in a CFRP composite layups	KNN SVM DT RF	Better DT performance with time domain features Better RF performance with frequency domain features
[151]	Data-driven	Damage classification in GFRP composites	Linear regression K-Means	The models correctly predict the type of damage, starting with both labelled and unlabeled data
[152]	Data-driven	Damage localization and quantification in a CFRP composite laminates	Bayesian algorithm Imaging algorithm	Good performance in detecting and visualizing damage
[153]	Model-based	Damage localization and quantification in composite structures	ANN	The network correctly predicts damage position and severity
[154]	Model-based	Damage quantification in a composite plate	YUKI-ANN	High accuracy and low computational time
[155]	Hybrid	Damage diagnosis in a CFRP composite plate and a composite beam	PCA SVM	SVM performance improves using NMB feature
[156]	Model-based	Damage detection, diagnosis of CFRP composite beams	Metropolis-Hastings algorithm	Working on raw data, reaching a high accuracy

**Table 8:** Summary of fatigue prognosis research articles.

Article	Approach	Scope	Algorithms	Results
[48]	Data-driven	Fatigue life estimation of GFRP composites	XGBoost LightGBM CatBoost K-Means	XGBoost prove to be the best algorithm. The results show an agreement between predicted and actual values
[69]	Data-driven	Health index evaluation of a carbon/epoxy composite plate	SDNN Bayesian	The extracted HIs meet the prognostic criteria
[70]	Data-driven	RUL prediction of composite panels	PCA, MLP Bayesian	RUL estimation is improved by considering a time-dependent model for HI evaluation
[157]	Data-driven	RUL evaluation of CFRP composite panels	GA GPR	The method is material-independent; it can be generalized to any SHM application
[158]	Data-driven	RUL evaluation of CFRP composite panels	GPR BNN	RUL estimation is more accurate when utilizing 10 features instead of 1; GPR requires less training time than BNN
[159]	Data-driven	RUL evaluation of multi-stiffened panels	GPR LSTM	The RUL estimation is close to the real one, but strongly depends on HIs
[160]	Data-driven	Health index evaluation of stiffened carbon/epoxy composite panels	PCA	HI3, HI4, vHI1, and vHI2 meet the Mo criterion, but not the Pr criterion. The two AE-based HIs meet the Pr criterion, but not the Mo criterion
[161]	Data-driven	Fatigue damage index prediction for CFRP composite coupons	ME KNN XGBoost NN	The proposed ME algorithm proves to be better than the others, and presents lower computational time
[162]	Data-driven	Fatigue damage diagnosis of a CFRP composite plate	DAE DBSCAN	The method does not require manual feature extraction; hence computation times are reduced
[163]	Data-driven	Fatigue damage mode clustering in CFRP open-hole laminated beams	AE + DIC correlation	When trained on AE data, the algorithm can predict the fatigue failure in real time, compared to DIC data

**Table 9:** Summary of impact research articles.

Article	Approach	Scope	Algorithms	Results
[164]	Data-driven	Impact detection and localization on a composite aircraft elevator	RF SAE ANN	Accuracy of 98.3%, 99.2%, and 96% for RF, SAE, and ANN
[165]	Data-driven	Impact localization in a composite aircraft elevator	Two SAE networks	Accuracy of 98% in classifying the impact zone; accuracy of 99.2% in localizing the impact
[166]	Data-driven	Impacts energy levels classification and uncertainty quantification in a composite plate	BNN ANN Multi-ANN	The algorithms can classify the energy levels and quantify the uncertainty for the perpendicular impacts Low performance arises for angle impacts
[167]	Data-driven	Localization of LVI in a composite plate	GA	Average localization error of 25.27 mm on a 500 × 500 mm plate
[168]	Data-driven	BVID recognition in a composite coupon	Four DL algorithm	ResNet best model to perform damage detection. Performance improves by embedding sensors within the structure
[169]	Data-driven	Impact localization and characterization in composite plates	CNN	The method accuracy is between 94.3% and 100% in predicting the impact location, while it is over 98.3% in classifying impact energy
[170]	Data-driven	Impact localization and characterization in CFRP composite plates	CNN	Accuracy of 99.76% and 97.04% for localization and characterization with MEMS data Accuracy of 99.58% and 98.68% for localization and characterization with PZT data
[171]	Data-driven	Impact localization and characterization in smart composite samples	CNN Bayesian optimization algorithm	Localization average error of 13.8% Classification average error of 11.3%
[172]	Data-driven	Impact localization in CFRP composite plate	Localization algorithm	The localization error for three drop heights, namely 50, 100, and 150 mm, is 8.8 mm, 9.5 mm, and 9.8 mm for a 300 × 300 mm plate
[173]	Data-driven	Impact localization in a composite plate	NSET ANN	The proposed method has an accuracy similar to that of the ANN-NSET method for hard impacts, while it is superior for soft impacts
[174]	Data-driven	Impact localization in flat and curved composite plates	NSET ANN	High accuracy of the ANN-NSET method, which is not affected by number of sensors and plate geometry
[175]	Model-based	Compression after impact strength prediction of carbon/glass hybrid laminates	XGBoost	Error of 3.55% in predicting the CAI strength

**Table 10:** Summary of other research articles.

Article	Approach	Scope	Algorithms	Results
[176]	Data-driven	Impact energy and damage detection on aerospace panels	Polynomial regression ANN	Successful regression for impact energy, classification for damage detection
[177]	Data-driven	Thin-walled structures, thin-walled shells	RNN (GRU-based)	High localization accuracy with limited sensors, end-to-end training
[178]	Model-based	Crack damage detection in a composite sandwich plate	GP	Best performance when employing three sensors With only one sensor, the accuracy is 100%, but misclassification can arise
[179]	Model-based	Crack length and orientation prediction of CFRP twill-woven plates	Polynomial regression ANN	Polynomial regression proves to be better than ANN
[180]	Model-based	Crack length evaluation of GFRP composite specimens	E-Jaya-ANN	The algorithm reaches the highest accuracy with eight hidden neurons
[181]	Data-driven	Crack identification in sandwich composites	SVM KNN DCNN	Accuracy of 78.23%, 85.56%, and 94.33% for SVM, KNN, and DCNN
[182]	Model-based	Classification of location and severity of cracks in GFRP composite beams	1D-CNN	Accuracy 95% and 93% for single/double damage classification With merged data to train the algorithm, accuracy of 92% after optimizing the hyperparameters
[183]	Data-driven	Crack classification in 3D woven composites	U-Net	Accuracy of more than 88% in crack classification
[184]	Data-driven	Matrix cracking classification in glass/epoxy laminates	SVM LVQ MLP	SVM and LVQ outperformed MLP, with SVM having the highest accuracy, at 91.7%
[185]	Model-based	Mechanical deformation, strength, and progressive damage prediction of a CFRP composite laminate with circular holes	ANN	Good performance and time reduction in global monitoring
[186]	Data-driven	Bending and torsional load spectra prediction on an aircraft wing	ARX, ARMAX, CFN, TDN, LRN, NXN LSTM, Bi-LSTM	Bending loads can be predicted by the auto-regressive models and ANNs, while torsional loads require the use of RNNs Tip torsional loads can be predicted only by Bi-LSTM
[187]	Data-driven	Real-time monitoring of NFRP composites during the machining phase	RF	RF shows better performance, with an accuracy of 94% in classifying cutting or non-cutting Accuracy of 95% in distinguishing fiber orientations towards the cutting orientation
[188]	Model-based	Optimization of number and orientation of sensors	NSGA-II GPR	The method can optimize the sensors layout The model is validated by conducting a delamination detection
[189]	Hybrid	Localization of acoustic sources in a composite laminate	BPN	Accuracy of 100% when trained on numerical data, while accuracy is lower when trained on experimental data
[190]	Hybrid	Breathing-debond assessment in fiber-reinforced stiffened composite panels	CNN CWT	Accuracy of 85.6% when trained on experimental data improved to 95.9% on training with higher harmonic signals

**Table 11:** Critical comparison of ML approaches for aerospace SHM tasks.

SHM Task	ML Approach	Strengths	Weaknesses/Challenges	Typical TRL
**Damage Detection**	Supervised Learning (SVM, RF, Decision Trees)	High accuracy with labeled data; interpretable in classical ML; robust for known damage modes	Requires large, labeled datasets; poor generalization to unseen damage types	4–6
	Unsupervised Learning (Clustering, PCA, Autoencoders)	No labels needed; good for novelty detection; adaptable to changing conditions	Higher false-positive rate; difficult to tune thresholds; less explainable	3–5
	Deep Learning (CNN, 1D/2D)	Learns directly from raw signals/images; handles complex patterns	High data/computational demand; “black-box” nature; certification barriers	3–5
**Damage Localization**	Model-Based ML (FEM + ML hybrid)	Incorporates physics; fewer experimental datasets needed; interpretable	Requires accurate structural model; sensitive to modelling errors	4–6
	Deep Learning (CNN, RNN, LSTM)	Captures spatial/temporal patterns; good for guided-wave data	Large training datasets needed; performance degrades under sensor drift	3–5
	Probabilistic Imaging + ML	Provides visual output; intuitive for operators	Dependent on sensor density/placement; computationally intensive	4–5
**Damage Classification and Quantification**	Ensemble Methods (RF, Gradient Boosting)	High accuracy; handles mixed feature sets well; robust to noise	Requires hand-crafted features; still needs labelled data	4–6
	Deep Neural Networks + Data Augmentation (GAN, DDPM)	Handles multiple damage types; reduces manual feature extraction	Synthetic data may not match flight conditions; heavy compute cost	3–5
	Hybrid PCA + ML	Reduces feature dimensionality; improves interpretability	May discard subtle features if poorly tuned	4–6
**Fatigue Prognosis/RUL Estimation**	Gaussian Process Regression (GPR)	Captures uncertainty; good for small datasets; interpretable	Limited scalability to very large datasets	4–6
	Recurrent Networks (LSTM, GRU)	Models time dependence; captures degradation trends	Needs long-term monitoring data; prone to overfitting	3–5
	Semi-supervised/Transfer Learning	Leverages small, labelled datasets; adaptable to new structures	Performance depends on domain similarity; not yet standardized in aerospace	2–4
**Multi-Task SHM**	Digital Twin + ML Hybrid	Combines simulation and real data; supports multiple SHM tasks	High setup cost; needs reliable real-time data integration	2–4

## Data Availability

Not applicable.
